# Functionally redundant Rho GTPases Cdc42 and RacA regulate aflatoxin synthesis and pathogenicity in *Aspergillus flavus* by controlling morphogenesis, oxidative balance and energy metabolism

**DOI:** 10.1080/21501203.2025.2527381

**Published:** 2025-07-10

**Authors:** Jia Xu, Junhe Ren, Yanyan Zhang, Zhuoyu Han, Qing Kong

**Affiliations:** School of Food Science and Engineering, Ocean University of China, Qingdao, China

**Keywords:** *Aspergillus flavus*, Cdc42, RacA, aflatoxin, pathogenicity, CRISPR/Cas9

## Abstract

Rho GTPases Cdc42 and RacA exhibit significant sequence homology and are conserved across eukaryotic species. These proteins function as molecular switches within various signal transduction pathways by cycling between GTP-bound (active) and GDP-bound (inactive) states. However, their specific functions in *Aspergillus flavus* remain largely unexplored. In this study, CRISPR/Cas9 system utilizing *5S rRNA* and tRNA-gRNA tandem arrays was developed, achieving over 95% single-gene and 75% double-gene editing efficiencies. Phenotypic and transcriptome analyses revealed significant functional redundancy between *cdc42* and *racA*. In the mutants, conidia germination was markedly accelerated, with early germination driven by increased hydrolase activity and ATP levels. The loss of either *cdc42* or *racA* resulted in reduced pathogenicity, compromised cell wall integrity, diminished aflatoxin production, and disrupted oxidative systems. These proteins regulate the generation of ROS through their interaction with NoxR, the regulatory subunit of NADPH oxidase (Nox). Cdc42 and RacA exhibited opposing roles in fatty acid β-oxidation and pyruvate metabolism. The simultaneous loss of function in both genes is lethal, as evidenced by the inability of the ∆*cdc42racA*_*tetOn*_ mutant to grow on plates lacking doxycycline. The study reveals the critical roles of the closely related genes *cdc42* and *racA* in the growth, development, and metabolism of *A. flavus*. These findings identify potential targets for mitigating the harmful effects of *A. flavus* and aflatoxins.

## GRAPHICAL ABSTRACT

**Figure:**
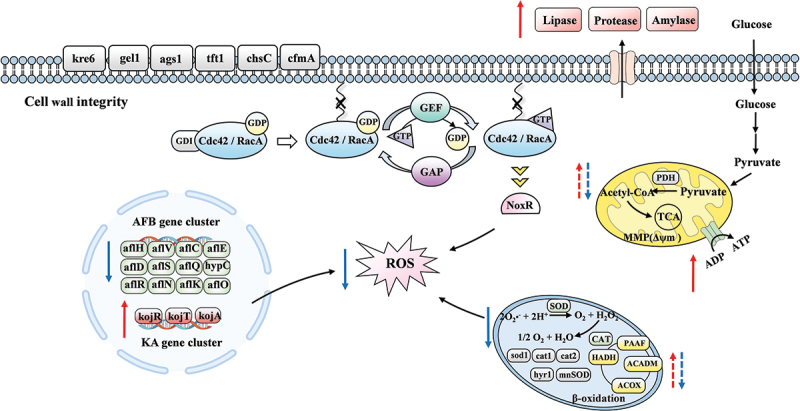

## 1. Introduction

As an opportunistic pathogen, *Aspergillus flavus* has a very broad host range (Klich 2007; Son and Park 2024). In the field, *A. flavus* primarily contaminates oil crops such as peanuts, cotton seeds, corn, and nuts (Zhao et al. 2024). However, under improper processing or storage conditions, it can grow on nearly all types of crop seeds, producing aflatoxins that contaminate food and feed (Xie et al. 2024b). *A. flavus* primarily produces aflatoxin B_1_ (AFB_1_) and aflatoxin B_2_ (AFB_2_), of which AFB_1_ is highly hepatotoxic, immunosuppressive, teratogenic, and mutagenic (Smela et al. 2001). In addition, *A. flavus* is a prevalent pathogen associated with aspergillosis, allergic rhinosinusitis, and fungal keratitis (Chowdhary et al. 2017). Given its profound impact on the economy, agriculture, environment, and human health, urgent efforts are needed to identify effective targets for inhibiting the reproduction of *A. flavus* and limiting aflatoxin contamination.

In recent years, extensive research has focused on the signal transduction pathways that regulate the pathogenicity of *A. flavus* and the biosynthesis of aflatoxin. The mitogen-activated protein kinase (MAPK) cascade represents a crucial phosphorylation pathway that transduces extracellular stimuli into corresponding cellular responses. The absence of *msg5* and *yvh1* phosphatases significantly diminishes aflatoxin production and the ability of *A. flavus* to infect crops (Yang et al. 2020). G protein-coupled receptors (GPCRs) are transmembrane receptors that facilitate signal transmission between intracellular and extracellular environments. They enable organisms to adapt to their surroundings and play essential roles in growth and development, stress factors, and signals derived from fatty acids (Affeldt et al. 2014). Rho GTPases belong to the Ras superfamily and function as molecular switches that regulate numerous signal transduction pathways in all eukaryotic cells (Etienne-Manneville and Hall 2002). They control complex cellular processes by cycling between two conformations (Hodge and Ridley 2016). Within the Rho GTPase family, Cdc42 and RacA show significant structural similarities and are key regulators that coordinate actin cytoskeleton organization and control cell polarity, but their functional deployment differs in filamentous fungi (Sordella et al. 2003; Virag et al. 2007). In *Aspergillus nidulans*, the lack of *racA* leads to developmental defects without impacting hyphal morphogenesis and Cdc42 is crucial for the timely formation of lateral branches (Virag et al. 2007). In the human pathogen *Candida albicans*, Cdc42 plays a crucial role in viability, hyphal growth, and pathogenicity. Cdc42 and Rac1 exhibit distinct kinetic properties on the membrane, and their functions are completely different (Bassilana and Arkowitz 2006). Contrary to filamentous fungi, *Saccharomyces cerevisiae* lacks RacA homologues, and Cdc42 is the main regulator of cell polarity. Multicellular filamentous fungi may require two Rho GTPases to regulate their greater morphogenic prowess (Harris 2011; Lichius et al. 2014), and the *racA* gene may have undergone a complex evolutionary process. In the rice fungal pathogen *Magnaporthe grisea* (Chen et al. 2008) and *A. flavus* (Qin et al. 2022), RacA is essential for virulence and pathogenicity, whereas in the human pathogen *Aspergillus fumigatus*, loss of RacA does not affect virulence but exhibits severe morphological defects (Li et al. 2011).

The CRISPR/Cas9 system is a universal genome editing technology with advantages such as simple manipulation, target specificity, efficient single/multiple-gene editing, and wide versatility (Jin et al. 2022). In this technology, a piece of RNA called single guide RNA (sgRNA) is produced, which recognizes the target DNA sequence and guides the expressed Cas9 for editing (Chang 2023). The sgRNA expression cassette can be controlled by a promoter recognized by RNA polymerase II (Pol II) or RNA polymerase III (Pol III). When using a type II promoter, it is essential to integrate hammerhead nuclease (HH) and hepatitis delta virus (HDV) nuclease at the 5‘and 3’ ends of the guide RNA (gRNA). This integration is necessary to eliminate modifications at both ends, thereby ensuring the proper execution of the guide and targeting functions (Kujoth et al. 2018; Shen et al. 2024). Type III promoters like *U6* and *U3* have also been developed, exhibiting 95% and 90% targeting frequencies in *A. flavus* (Chang 2023) and *A. nidulans* (Nødvig et al. 2018), but they show base preference during transcription, reducing the number of available targets. The endogenous *5S rRNA* promoter in *A. niger* proposed by Zheng et al. (2018) has the advantages of high conservation, well-defined structure, and efficient transcription by Pol III, achieving a gene editing rate exceeding 96%. This study identified and utilized the *5S rRNA* gene promoter to regulate the sgRNA expression cassette in *A. flavus* for the first time, achieving single-gene editing efficiency exceeding 95% and successfully knocked out the *cdc42* and *racA* genes. RNase P and RNase Z use the tRNA splicing mechanism to release mature tRNAs from larger pre-tRNA transcripts (Phizicky and Hopper 2010). Using the endogenous tRNA-gRNA tandem array system, two sgRNAs were co-expressed within a single genome editing plasmid, achieving a double-gene editing efficiency exceeding 75%. However, no *cdc42/racA* double-gene mutants were obtained, suggesting that the simultaneous deletion of *cdc42* and *racA* is lethal. This conclusion was further validated through the generation of a doxycycline-regulated ∆*cdc42racA*_*tetOn*_ mutant, which exhibited no growth on plates in the absence of doxycycline.

Our study demonstrated that *cdc42* and *racA* function redundantly and co-regulate the growth, aflatoxin biosynthesis, pathogenicity, and oxidative systems of *A. flavus*. These findings uncover a novel regulatory mechanism involving *cdc42* and *racA* and highlight potential targets for controlling *A. flavus* infections and reducing aflatoxin contamination.

## 2. Materials and methods

### 2.1. Strains and culture conditions

The *A. flavus* strains used and constructed in this study are detailed in Table S1. The wild type (WT) *A. flavus* NRRL3357 was used for evaluating the efficiency of CRISPR/Cas9 system and constructing mutants. The strains were incubated on potato dextrose agar (PDA), glucose minimal medium (GMM), and yeast extract medium (YES) at 30 °C for several days and used for subsequent analysis. *A. flavus* strains were spotted on GMM medium supplemented with various stress inducers. Osmotic stress was induced using 1 mol/L NaCl, oxidative stress with 5 mmol/L or 10 mmol/L H₂O₂, cell wall stress with 10 μg/mL or 50 μg/mL Congo red (CR) and 50 μg/mL or 100 μg/mL sodium dodecyl sulfate (SDS), and thermal stress by incubation at 40 °C.

### 2.2. Construction of gene knockout and complementation strains

**Figure 1.**
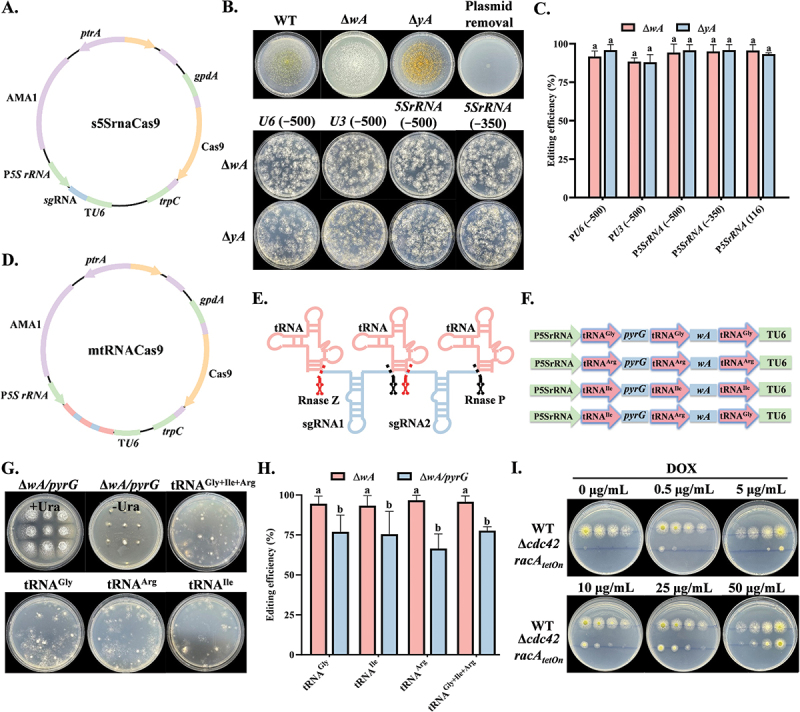
Mutant construction and gene-editing efficiency analysis. (A) Construction of a genome-editing plasmid driven by the *5S rRNA* promoter. (B) Phenotypes of Δ*wA* (white conidia) and Δ*yA* (yellow conidia) mutants generated using different promoters for sgRNA expression. Plasmids were removed after two passages on plates without selection pressure. (C) Editing efficiency of the 5SrRNA-Cas9 genome-editing plasmid. (D) Construction of a genome-editing plasmid based on the tRNA-gRNA tandem array system. (E) Mechanism of action of the tRNA-gRNA tandem array system. (F) Design of the tandem array system mediated by glycine tRNA, arginine tRNA, and isoleucine tRNA in *Aspergillus flavus*. (G) Phenotypes of Δ*wA*/*pyrG* double-gene mutants grown on plates with and without uracil supplementation. (H) Editing efficiency of the mtRNA-Cas9 genome-editing plasmid. (I) Serial 10-fold dilutions of the WT and Δ*cdc42racA*_*tetOn*_ strains were spotted on GMM medium supplemented with varying concentrations of doxycycline and incubated at 30 °C for 48 h. The error bars represent the standard errors, and different letters above the bars represent significantly different values (*p <* 0.05).

The *A. flavus 5S rRNA* gene sequence was sourced from the *5S rRNA* database (https://rnacentral.org/). The *U6* terminator and its flanking scaffold sequence were amplified from the pAf-CRISPR-yA plasmid (Addgene plasmid #191015) using primer pair TU6-F/scaffold-R. The *5S rRNA* gene, including its internal promoter and its upstream partial sequence *5S rRNA* (−X) and protospacer, was synthesized by Sangon Biotech and amplified using primer pair P5SrRNA-F/5SrRNA-R, with the forward primer containing an 18-bp overlap sequence. The two fragments were fused and cloned into the pAf-CRISPR-yA plasmid digested with *Kpn*I and *Pst*I to obtain the single gene editing plasmid s5SrnaCas9 (Figure 1A). The *5S rRNA* (−500) and *5S rRNA* (−350) sequences were selected for synthesis and assembled using a consistent methodology. The *wA* and *yA* genes were selected to assess the efficiency of different lengths of *5S rRNA* promoters, *U3* promoter, and the *U6* promoter. The transformation of *A. flavus* followed the procedure described by Chang (2023), and the number of transformants was quantified in three independent transformation experiments. Single-gene mutants targeting *cdc42* and *racA* were generated using the above plasmids. Two CRISPR RNAs (crRNAs) were designed using Cas-Designer (http://www.rgenome.net/cas-designer/) to minimize off-target mismatches. The corresponding sequences were presented in Table S1, and transformants were selected in two independent transformation experiments. The genome-editing plasmids were removed by passage twice on antibiotic-free plates, and a 1.0 kb DNA fragment surrounding the target sequence was amplified using primer pairs cdck-F/cdck-R and rack-F/rack-R to investigate gene deletions. The *ptrA* selection marker was used repeatedly to obtain complementation strains by homologous recombination (HR), and the results were verified by PCR. All primers used in this study are listed in Table S2.

The tRNA gene sequences used in this study were sourced from the *A. flavus* NRRL3357 genome database in NCBI (https://www.ncbi.nih.gov/), and three amino acids with high content, glycine, isoleucine, and arginine, were selected. These sequences, along with the 76-bp scaffold sequence were used to construct the sgRNA expression vector. The tandemly arranged tRNA-sgRNA*wA*-tRNA-sgRNA*pyrG*-tRNA sequence and the 116-bp *5S rRNA* gene sequence were synthesized by Sangon Biotech and then cloned into the pAf-CRISPR-yA plasmid digested with *Kpn*I and *Pst*I to obtain the multi-gene editing plasmid mtRNACas9 (Figure 1D). The *wA* and *pyrG* genes were selected to assess the efficiency of various tRNA-gRNA arrays in three independent transformation experiments. The sgRNAs of *cdc42* and *racA* were cloned into the mtRNACas9 plasmid to construct double-gene mutants (∆*cdc42*/*racA*). To determine whether the simultaneous loss of *cdc42* and *racA* gene functions is lethal, we constructed a doxycycline-inducible ∆*cdc42racA*_*tetOn*_ mutant. The Tet-On promoter sequence was chemically synthesized by Sangon Biotech (Shanghai, China).

### 2.3. Growth, sclerotia and conidia analysis

To analyze colony growth, *A. flavus* strains were spotted on GMM and YES media and incubated at 30 °C in the dark for 4 d. Vegetative colony growth was assessed by measuring colony diameter. For the sclerotia assay, *A. flavus* strains were spotted on GMM medium supplemented with 2% sorbitol and incubated at 30 °C in the dark for 7 d. To visualize sclerotia morphology, 75% ethanol was sprayed onto each plate, and the number of sclerotia in different areas was observed and counted. To evaluate conidiophore morphology, strains were cultured on coverslips placed on GMM medium for 3 d and processed using the method described by Yang et al. (2017). *A. flavus* strains were spotted on YES, GMM, and PDA media, and conidia formation was analyzed after incubation for 6 d. For conidia germination rate analysis, 10^7^ spores/mL suspension was spread on GMM medium. The conidia germination was observed at 6, 8, 10, and 12 h, and the germination rate was calculated. This experiment was carried out in triplicate.

### 2.4. Fluorescence microscopy observation

A suspension containing 10^6^ spores/mL was inoculated into PDB medium and incubated in a shaker at 30 °C and 180 r/min for 16 h. To visualize nuclei, the harvested mycelia were stained with 10 μg/mL Hoechst 33258 (Beyotime, Haimen, China). Sterol-rich membrane regions were stained using 50 μg/mL filipin (Sigma, Shanghai, China). Reactive oxygen species (ROS) content was assessed by staining the mycelia with 10 μmol/L DCFH-DA (Beyotime, Haimen, China). Lipid droplets (LDs) were visualized with 10 μg/mL Nile red. For septa and chitin deposition, the mycelia were stained with 20 μg/mL calcofluor white (CFW) (Sigma, Shanghai, China). Additionally, β-1,3-glucan content was detected by staining the mycelia with 500 μg/mL aniline blue (Solarbio, Beijing, China). After staining, the mycelia were washed three times with PBS and observed under a fluorescence microscope (Nikon, Tokyo, Japan).

For the detection of chitin and mannan, 10^7^ conidia were labeled with WGA-FITC and ConA-FITC (Sigma, Shanghai, China), respectively. To assess mitochondrial membrane potential (Δψm), JC-1 was used as a fluorescent probe, and the samples were stained with a mitochondrial membrane potential determination kit (Solarbio, Beijing, China). After staining, the conidia were washed three times with PBS and subsequently observed and quantified using a fluorescence microscope and a flow cytometer (BD Biosciences, USA).

### 2.5. Aflatoxin (AF), kojic acid (KA), and cyclopiazonic acid (CPA) analysis

*A*. *flavus* strains were spotted on GMM and YES media at 30 °C in darkness for 7 d. The AFB content was assessed using the method outlined by Wang et al. (2022). Three agar plugs, each with a diameter of 1.5 cm, were taken from each plate and transferred to a 10 mL centrifuge tube. Subsequently, 5 mL of methanol was added, and the mixture was vortexed for 30 min and shaken at 200 r/min for 2 h to extract the aflatoxins. The AFB_1_ ELISA kit (Jiangsu Huisi Technology Co., Ltd., Jiangsu, China) was employed for quantification. *A. flavus* strains were spotted on YES medium at 30 °C for 7 d, and the CPA content was measured using the previously described method (Xu et al. 2025). Three agar plugs, each with a diameter of 1.5 cm, were obtained from each plate and transferred to a 10 mL centrifuge tube. Subsequently, 5 mL of chloroform was added, and the mixture was vortexed for 30 min and shaken at 200 r/min for 2 h to extract CPA. KA reacts with Fe^3+^ to form a red-colored compound. *A. flavus* strains were spotted on kojic acid medium (KAM) at 30 °C for 5 d, and the KA content was visually assessed. For quantitative analysis, a 10^7^ spores/mL suspension was inoculated into KAM, and the KA content in the supernatant was measured by colorimetry on days 5 and 7. All experiments were conducted in triplicate.

### 2.6. Seed infection and surface adhesion ability analysis

Peanut and corn kernels were surface-sterilized using 75% ethanol following a previously described method (Xu et al. 2025). Ten grams of seeds were placed in a 250 mL flask, inoculated with 1 mL of a spore suspension (10^5^ spores/mL), and incubated at 30 °C under 90% relative humidity. After 3 d, contaminated peanut seeds were sectioned using a razor blade, fixed overnight in 2.5% glutaraldehyde, and imaged with a JEM-1200EX electron microscope. After 7 d, the conidia produced on the infected seeds were quantified, and the AFB content was analyzed using TLC. The experiment was conducted in triplicate.

A total of 100 µL of 10^6^ spores/mL suspension was added to the wells of a sterile 96-well polystyrene plate, with 24 replicates per strain. The cultures were incubated at 30 °C for 48 h and 72 h. The surface adhesion ability of *A. flavus* was assessed following the method described by Lohmar et al. (2019).

### 2.7. Hydrogen peroxide (H_2_O_2_), malondialdehyde (MDA), antioxidant enzyme activity (SOD and CAT), adenosine triphosphate (ATP), and acetyl coenzyme A (acetyl-CoA) assay

A total of 10^6^ conidia were inoculated into 100 mL of PDB medium and cultured at 30 °C with shaking at 180 r/min for 36 h. The harvested mycelia were dried, and approximately 0.1 g of fungal tissue was weighed and ground in liquid nitrogen. The levels of H₂O₂ and MDA, as well as the activities of CAT and SOD, were measured using detection kits (Solarbio, Beijing, China). A total of 10^6^ conidia were inoculated into 100 mL of YES medium and cultured at 30 °C with shaking at 180 r/min for 72 h. Tissue homogenates were prepared as previously described, and acetyl-CoA and ATP content were measured using an acetyl-CoA assay kit and an ATP assay kit (Solarbio, Beijing, China), respectively.

### 2.8. Yeast two-hybrid (Y2H) assay

The coding sequences (CDS) of the *racA, cdc42*, and *noxR* genes were amplified and inserted into the plasmids pGADT7-AD and pGBKT7-BD. These plasmids were transformed into the AH109 strain and plated on 2DO (SD/-Leu/-Trp) plates. The transformants were further streaked onto selective 4DO (SD/-Leu/-Trp/-His/-Ade) plates and subsequently spotted onto SD/-Leu/-Trp/-His/-Ade/X-α-Gal plates. All selective media and reagents used in this experiment were obtained from Solarbio (Beijing, China).

### 2.9. Lipid utilization and hydrolytic activity assay

Lipid utilization was evaluated using MM medium supplemented with 50 mmol/L sodium acetate, 2.5 mmol/L palmitic acid, 2.5 mmol/L oleic acid, and 5% Tween-20. Growth was assessed by measuring the colony diameter after 5 days of cultivation, and each strain was tested in triplicate.

To analyze amylase activity, *A. flavus* strains were inoculated on AMM medium containing 0.5% soluble starch and incubated at 30 °C in the dark for 3 d. Lipase activity was assessed by culturing the strains on MM medium supplemented with 2.5 mmol/L palmitic at 30 °C in the dark for 5 d. The relative degradation rate was calculated as the ratio of the outer circle diameter (clear zone) to the inner circle diameter (fungal colony diameter). Protease activity was induced following the method described by Duran et al. (2014). After 48 h, the supernatant was collected and measured using the azocasein assay.

### 2.10. Quantitative reverse transcription-PCR and transcriptome analysis

A spore suspension containing 10^8^ spores/mL was inoculated into PDB and incubated at 180 r/min for 13 h. Mycelia were collected by sterile Miracloth and plated on PDA medium, and samples were taken at 12 and 72 h. Total RNA was extracted using the Fungal RNA Kit (Omega BioTek, Darmstadt, Germany). RT-qPCR was performed in a 20 μL reaction volume according to previously described protocols (Xu et al. 2024a). The *A. flavus* 18S rRNA gene served as an internal reference for normalization, and relative gene expression was calculated using the 2^−ΔΔCt^ method.

cDNA libraries were constructed according to the Illumina RNA-seq library method by Shanghai Meiji Biomedical Technology (Shanghai, China) and sequenced on an Illumina Novaseq 4000. The expression level of each gene was calculated by quantifying the cDNA fragments per kilobase of transcripts per million fragments mapped (TPM) using Cufflinks software. Expression profiles in genes and transcripts were quantified by Salmon expression quantification software (Patro et al. 2017). Genes were considered significantly differently expressed genes (DEGs) when |log2(fold change)| > 1 and the -Log10(Padjust) > 1.3. Data can be accessed from NCBI SRA (BioProject ID: PRJNA1181790). To further reveal the functions of the DEGs, enrichment analysis was conducted using the gene ontology (GO) function and the Kyoto Encyclopedia of Genes and Genomes (KEGG) pathway.

### 2.11. Bioinformatic analysis

The genes and amino acid sequences of *A. flavus* Cdc42 (NCBI accession number XM_002382143.1) and RacA (NCBI accession number XM_002384111.1) were obtained from NCBI (https://www.ncbi.nlm.nih.gov/). Homologues in other eukaryotic species were identified using BLASTP and Uniprot (http://www.uniprot.org/) search tools, and the phylogenetic tree was constructed using MEGA11.0 software. All sequences were aligned with the ClustalW tool (https://www.genome.jp/tools-bin/clustalw), and the conserved regions were analyzed using Jalview software.

### 2.12. Statistical analysis

The data were analyzed using IBM SPSS Statistics 25. Analysis of variance (ANOVA) and Tukey’s multiple comparison test were used to compare differences between groups. Statistical significance was considered at *p* < 0.05.

## 3. Results

### 3.1. *Construction of mutants based on* 5S rRNA *promoter and tRNA*

The highly conserved and abundant *5S rRNA* allows transcription by RNA polymerase III through its internal gene promoter (Figure 1A). In this study, we identified the *5S rRNA* gene sequence (Figure S1) and truncated the upstream 5’ region (−500 and −350) containing unknown functional elements to target the *wA* and *yA* genes. After transformation, transformants with the expected phenotype were obtained, forming yellow and white conidia, and the plasmid containing the AMA1 sequence could be lost after two passages on antibiotic-free plates (Figure 1B). We found that the editing efficiency based on the *5S rRNA* promoter was not affected by the truncation of the upstream unknown functional elements and was as efficient as *U6* promoter. The 5*S rRNA* with a length of only 116-bp could well initiate the expression of sgRNA, with editing efficiency of 95.33% for the *wA* and 93.27% for the *yA* (Figure 1C). The optimized CRISPR/Cas9 system was used to target the *cdc42* and *racA* genes, and positive transformants were preliminarily selected by morphological observation. The primer pairs cdck-F/cdck-R and rack-F/rack-R were used for amplification and sequencing (Figure S2A), and deletions of different lengths were detected in ∆*cdc42* (1 bp, 5 bp) and ∆*racA* (1 bp, 9 bp). Subsequently, ∆*cdc42*-1 and ∆*racA*-1 were selected for the construction of complementation strains (Figure S2B), and PCR verified that the *cdc42* and *racA* complementation strains were successfully constructed (Figure S2C).

Multiple mutagenesis in a single transformation step can significantly enhance gene editing efficiency. tRNA is abundant in cells, and its internal promoter element serves as a potential transcription enhancer of Pol III (Xie et al. 2015). Within the nucleoplasm, RNase P and RNase Z cleave the pre-tRNA to eliminate the extra sequences at 5′ and 3′ ends (Figure 1D,E). Therefore, we tried to use the endogenous tRNA-gRNA tandem arrangement system to simultaneously express two sgRNAs in a single genome editing plasmid and identified the tRNA gene sequences for glycine, arginine, and isoleucine in *A. flavus* (Figure S1). We constructed plasmids that simultaneously targeted the *wA* and *pyrG* genes to test the efficiency of this system (Figure 1F). White transformants formed by the deletion of the *wA* gene could be observed on the primary transformation plate. In the subsequent round of testing, the double-gene mutants were unable to grow on uracil-deficient plates (Figure 1G). Due to the inherent high efficiency of the *wA* gene target site, its editing efficiency has been consistently maintained at nearly 95%. For double-gene knockout, we found that the editing efficiency of the system mediated by glycine and isoleucine tRNA was notably high at 76.95% and 75.51%, and the editing efficiency of arginine tRNA was the lowest at 66.47% (Figure 1H). Sequencing analysis revealed that there were insertions or deletions at the *pyrG* target site (Figure S2D). To avoid potential instability of the sgRNA expression cassette from using identical tRNA sequences (Li et al. 2021), we arranged the tRNAs of the three amino acids in tandem to enhance plasmid stability, resulting in a single gene editing efficiency of 95.69% and a double-gene editing efficiency of 77.59% (Figure 1H).

Previous studies have revealed that the simultaneous loss of *cdc42* and *racA* function is lethal in *A. niger* (Kwon et al. 2011) and *A. nidulans* (Virag et al. 2007). To investigate whether this phenomenon also occurs in *A. flavus*, we utilized the multi-gene editing system described above to generate Δ*cdc42*/*racA* mutants. Among 30 verified transformants, the generation rates of single-gene mutants for *cdc42* and *racA* were 47.92% and 32.62%, respectively. However, no double-gene mutants were obtained (Table S1). Furthermore, the Δ*cdc42racA*_*tetOn*_ mutant exhibited colony development only in the presence of doxycycline (Figure 1I). These findings indicate that the simultaneous loss of *cdc42* and *racA* function is lethal in *A. flavus.*

### 3.2. *Cdc42 and RacA are closely related in* A. flavus

Cdc42 (AFLA_129910) and RacA (AFLA_102810) have high structural similarity (identity 64.2%, similarity 77.4%), with the classic TQXD sequence in Cdc42 located at amino acid positions 117 − 120, and the TKLD in RacA located at 126 − 129 (Figure S3). Both proteins belong to the Rho subfamily within the GTPase Ras superfamily, and their homologues are also widely present in eukaryotic genomes. Bioinformatics analysis revealed that the Cdc42 and RacA proteins in *A. flavus* exhibited high conservation levels with *A. fumigatus* and *Penicillium digitatum*, while showing relatively lower conservation with *C. albicans*. Notably, no homologue of RacA have been identified in *S. cerevisiae* (Table S3). These homologues all contain a GTP binding/hydrolysis domain (red box), an effector domain (black box), a membrane localization motif (blue box), and a Rho insertion domain (yellow box) (Figure S3).

### 3.3. *Effects of* cdc42 *and* racA *on phenotype*

**Figure 2.**
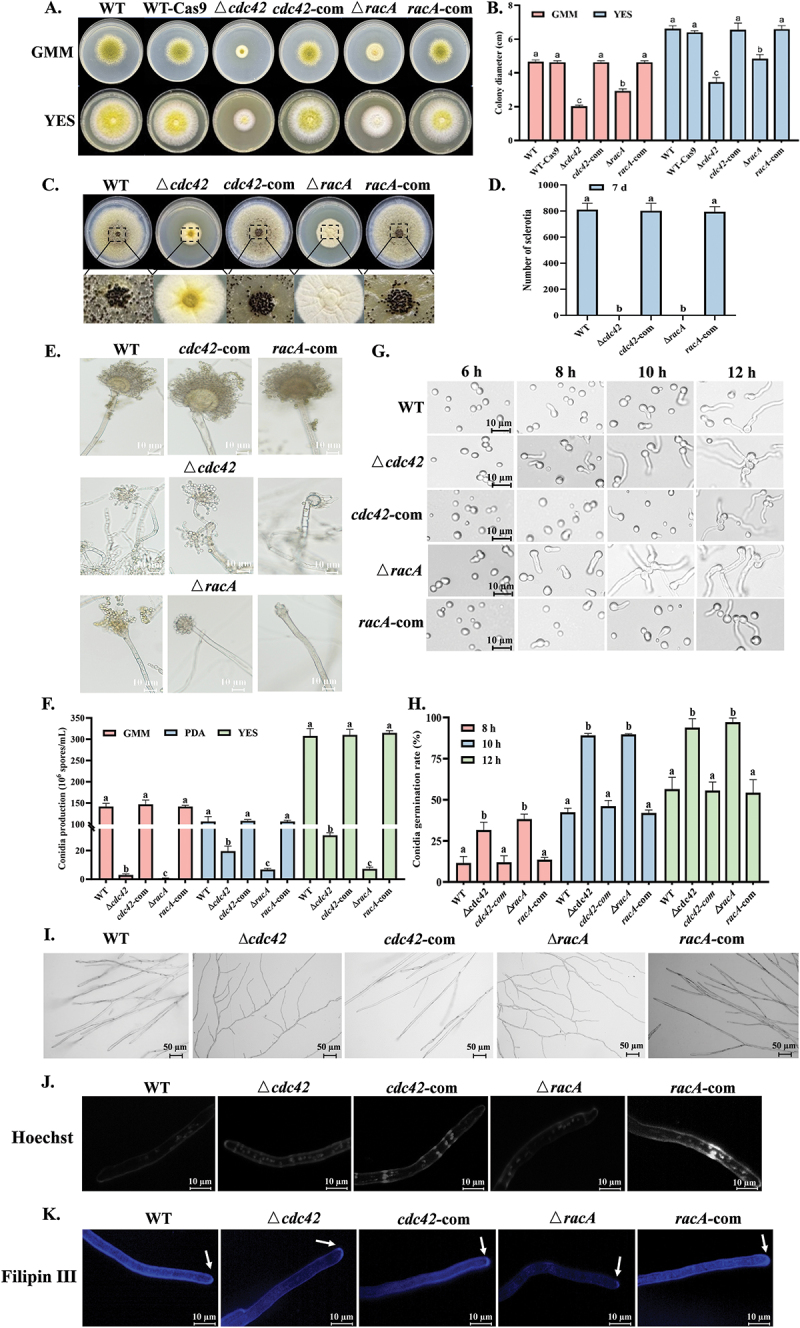
Effects of *cdc42* and *racA* on growth, sclerotia, conidia, and hyphae. (A) Colony morphology of *Aspergillus flavus* strains after 4 d of cultivation on PDA and YES medium at 30 °C. (B) Colony diameter of all strains. (C) Sclerotia morphology of all strains after 7 d of cultivation on GMM medium containing 2% sorbitol at 30 °C. (D) Sclerotia production of all strains. (E) Conidia morphology of all strains after 72 h of cultivation on GMM medium at 30 °C. (F) Conidia production of all strains after 4 days of cultivation on GMM, PDA, and YES medium at 30 °C. (G) Germination of all strains after 6, 8, 10, and 12 h of cultivation on GMM medium at 30 °C. (H) Conidia germination rate of all strains after 8, 10 and 12 h of cultivation on GMM medium at 30 °C. (I) Microscopic observation of hyphae after 48 h of cultivation on GMM medium at 30 °C. (J) Distribution of nuclei in strains after 15 h of growth in PDB medium at 30 °C. (K) Distribution of sterols at hyphal tips in strains after 15 h of growth in PDB medium at 30 °C. The error bars represent the standard errors, and different letters above the bars represent significantly different values (*p <* 0.05).

When using mutants generated by the CRISPR/Cas9 system to study gene function, it is critical to assess whether the expression of the Cas9 protein affects the growth (Fuller et al. 2015). After transformation of *A. flavus* with the Cas9 protein plasmid without sgRNA, there was no significant difference compared with the WT (Figure 2A). Compared to the WT and the complementation strain, the mutants exhibited reduced radial growth rates, with the ∆*cdc42* mutant displaying the smallest colony diameter (Figure 2A,B). Our observation that the mutants were unable to produce sclerotia emphasized the key role of *cdc42* and *racA* in the development of *A. flavus* sclerotia (Figure 2C,D). Microscopic observation of conidial morphology revealed that the ∆*racA* mutant produced the fewest conidia, consistent with previous test results. Additionally, the mutants exhibited slender stipes, irregularly shaped vesicles, and reduced numbers of sterigmata (metulae and phialides), and conidiospores (Figure 2E,F). Interestingly, the conidial germination rate of the mutants was higher than that of the wild type. At 6 h, the roundness of the ∆*racA* conidia decreased, and germ tubes emerged, indicating earlier dormancy breaking. By 10 h, the germination rates of ∆*cdc42* and ∆*racA* were both close to 90%, whereas the WT reached only 42.39%. These results suggest that *cdc42* and *racA* play a negative regulatory role in the conidial germination process (Figure 2G,H). Cdc42 and RacA are essential for maintaining the cytoskeleton. In WT strain, hyphae were plump and straight, whereas the tips of hyphae in ∆*cdc42* and ∆*racA* strains were curled and exhibited frequent branching, indicating a loss of polarity (Figure 2I). Nuclear distribution analysis revealed that the size and shape of nuclei in the mutants were comparable to those in the WT and complementation strains. However, the ∆*cdc42* mutant exhibited a significant increase in the number of nuclei per unit hyphal length, suggesting that while the cytoplasmic volume of ∆*cdc42* remained normal, its hyphal elongation rate was reduced (Figure 2J). Ergosterol, a key component of the sterol-rich plasma membrane domain (SRD) at hyphal tips, plays a critical role in maintaining growth polarity (Xie et al. 2024a). In the WT and complementation strains, concentrated fluorescence at the hyphal tips formed a cap-like structure (white arrow), a pattern also observed in ∆*cdc42*. However, the ∆*racA* mutant lost ergosterol accumulation at the hyphal tips, accompanied by an overall reduction in ergosterol content (Figure 2K).

### 3.4. *Effects of* cdc42 *and* racA *on secondary metabolism and pathogenicity*

**Figure 3.**
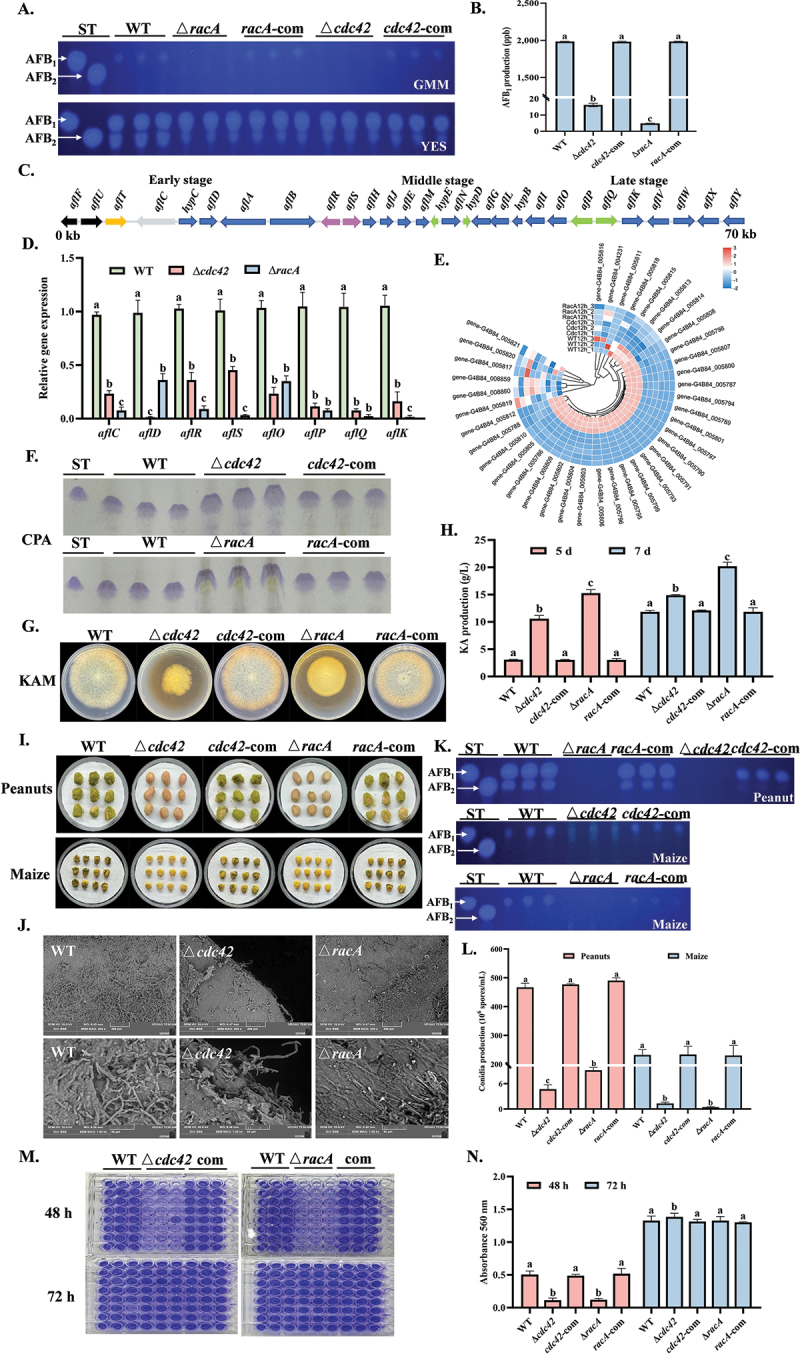
Effects of *cdc42* and *racA* on secondary metabolite and pathogenicity in *Aspergillus flavus*. (A) TLC was used to analyze AFB levels in all strains after 7 d of cultivation on GMM and YES media at 30 °C. (B) Aflatoxin content was quantified using an ELISA kit after 7 d of cultivation on GMM at 30 °C. (C) A schematic diagram of the aflatoxin biosynthesis gene cluster. (D) The RT-qPCR analysis of genes related to AFB synthesis. The expression of *18S rRNA* was used as an internal reference. (E) RNA-seq analysis of the effects of *cdc42* and *racA* on the expression profiles of genes involved in the biosynthesis of secondary metabolites. (F) TLC was used to measure CPA levels in all strains after 7 d of cultivation on YES medium at 30 °C. ST: CPA standard. (G) The reactions of strains to Fe^3+^ were observed after 7 d of cultivation on GMM at 30 °C. (H) KA production was assessed in KAM medium after 5 and 7 d of cultivation at 30 °C. (I) All strains were inoculated on seeds and incubated at 30 °C for 7 d. (J) TEM was used to observe *A. flavus* colonization on peanut surfaces after 3 d. (K) TLC was used to analyze aflatoxin levels in all strains after 7 d of cultivation on peanuts at 30 °C. (L) Conidia production was quantified after 7 d of infection on peanuts. (M, N) Adhesion and absorbance of all strains were measured after 48 h and 72 h of cultivation at 30 °C. The error bars represent the standard errors, and different letters above the bars represent significantly different values (*p <* 0.05).

Aflatoxin is among the most concerning secondary metabolites produced by *A. flavus* due to its high carcinogenic and teratogenic potential. On GMM medium, trace amounts of aflatoxin were detected in the mutants, with aflatoxin levels in the ∆*cdc42* and ∆*racA* mutants reduced by 120-fold and 400-fold, respectively, compared to the WT (Figure 3A,B). Transcriptome analysis revealed that 21 genes involved in the early, middle, and late stages of aflatoxin synthesis were significantly downregulated, a finding further confirmed by RT-qPCR (Figure 3C−E). However, in nutrient-rich medium, aflatoxin production was unaffected, likely due to the activation of alternative signaling pathways bypassing the dependence on *cdc42* and *racA* (Figure 3A). We also observed similar phenomena in the production of CPA, a key pathogenic factor of the saprophytic lifestyle of *A. flavus* (Figure 3E,F). KA is a potent antioxidant that can scavenge ROS and chelate with Fe^3+^ to form red compounds (Zhang et al. 2014), the levels of which are negatively correlated with aflatoxin levels (Figure 3G). The results indicated that KA content in the mutants increased significantly on the 5th and 7th days (Figure 3H). Transcriptome analysis revealed the upregulation of genes associated with KA synthesis, including *kojA*, *kojR*, and *kojT* (Figure 3E). The loss of *cdc42* and *racA* significantly impaired the colonization ability and pathogenicity of *A. flavus*. TEM revealed that the hyphae of the WT were thick and solid, completely covering the surface of peanuts, whereas the hyphae of the mutants appeared thin and collapsed, resulting in severely reduced colonization ability (Figure 3I,J). Compared to the WT, the number of conidia produced by the ∆*cdc42* and ∆*racA* mutants decreased by 100-fold and 50-fold, respectively, and aflatoxin production was undetectable (Figure 3K,L). Furthermore, the mutants exhibited reduced adhesion during the critical early stages of infection (Figure 3M,N).

### 3.5. *Effects of* cdc42 *and* racA *on osmotic and oxidative stress responses*

**Figure 4.**
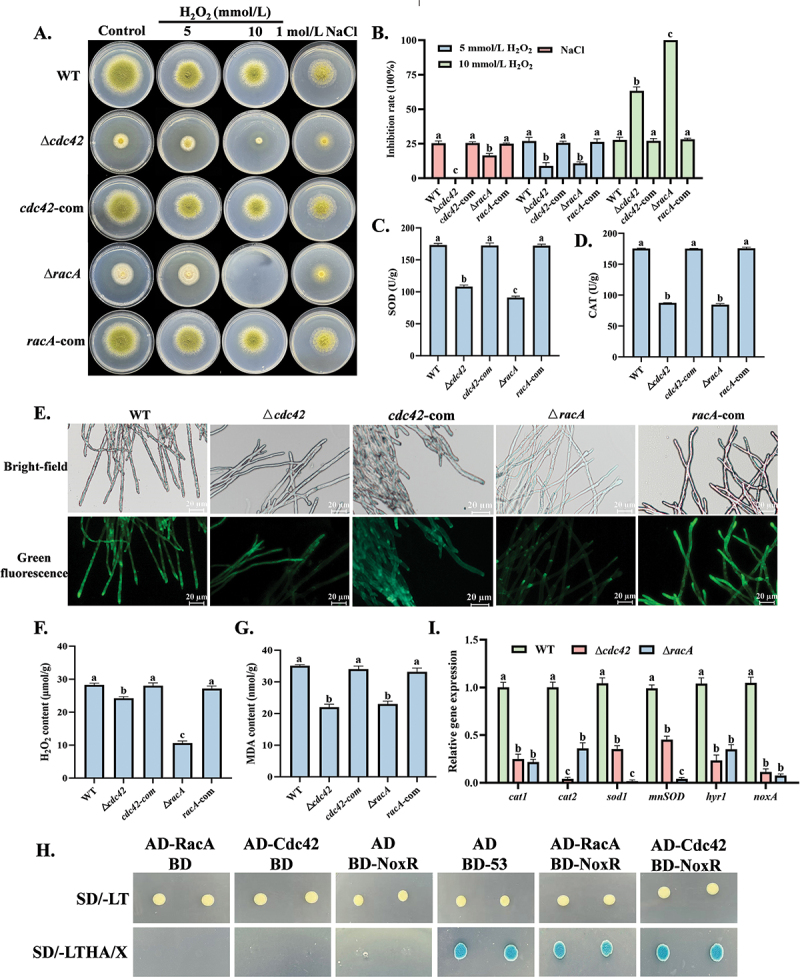
Effects of *cdc42* and *racA* on osmotic and oxidative stress. (A) Colony morphology of all strains cultured on GMM medium supplemented with H_2_O_2_ and NaCl at 30 °C for 5 d. (B) Growth inhibition rates were calculated based on colony diameters. (C, D) Enzyme activities of SOD and CAT in all strains after cultivation at 30 °C for 36 h. (E) ROS content in all strains was analyzed using fluorescence microscopy. (F, G) H₂O₂ and MDA content in all strains after cultivation at 30 °C for 36 h. (H) Interaction between Cdc42/RacA and NoxR was verified using Y2H analysis. (I) RT-qPCR analysis of genes associated with oxidative stress. The error bars represent the standard errors, and different letters above the bars represent significantly different values (*p <* 0.05).

In osmotic and oxidative stress tests, the deletion of *cdc42* and *racA* significantly increased the sensitivity of *A. flavus* to oxidative stress (Figure 4A). When exposed to 10 mmol/L H₂O₂, the inhibition rate of the Δ*cdc42* reached 63.3%, while the Δ*racA* exhibited complete growth inhibition (Figure 4A,B). Additionally, the activities of SOD and CAT, which are essential for scavenging ROS, were significantly reduced in the mutants (Figure 4C,D). Assays measuring ROS revealed that the mutants exhibited significantly lower ROS levels compared to the WT, with the Δ*racA* mutant demonstrating the lowest levels of ROS (Figure 4E), a finding that was further corroborated by flow cytometry analysis (Figure S4). This reduction was accompanied by a decrease in the levels of H₂O₂ and MDA (Figure 4F,G). Furthermore, yeast two-hybrid assays confirmed the interaction between RacA/Cdc42 and the regulatory subunit NoxR of NADPH oxidase (Nox) (Figure 4H). Additionally, the deletion of *cdc42* and *racA* downregulated the expression of genes associated with oxidative stress (Figure 4I).

### 3.6. *Effects of* cdc42 *and* racA *on cell wall integrity*

**Figure 5.**
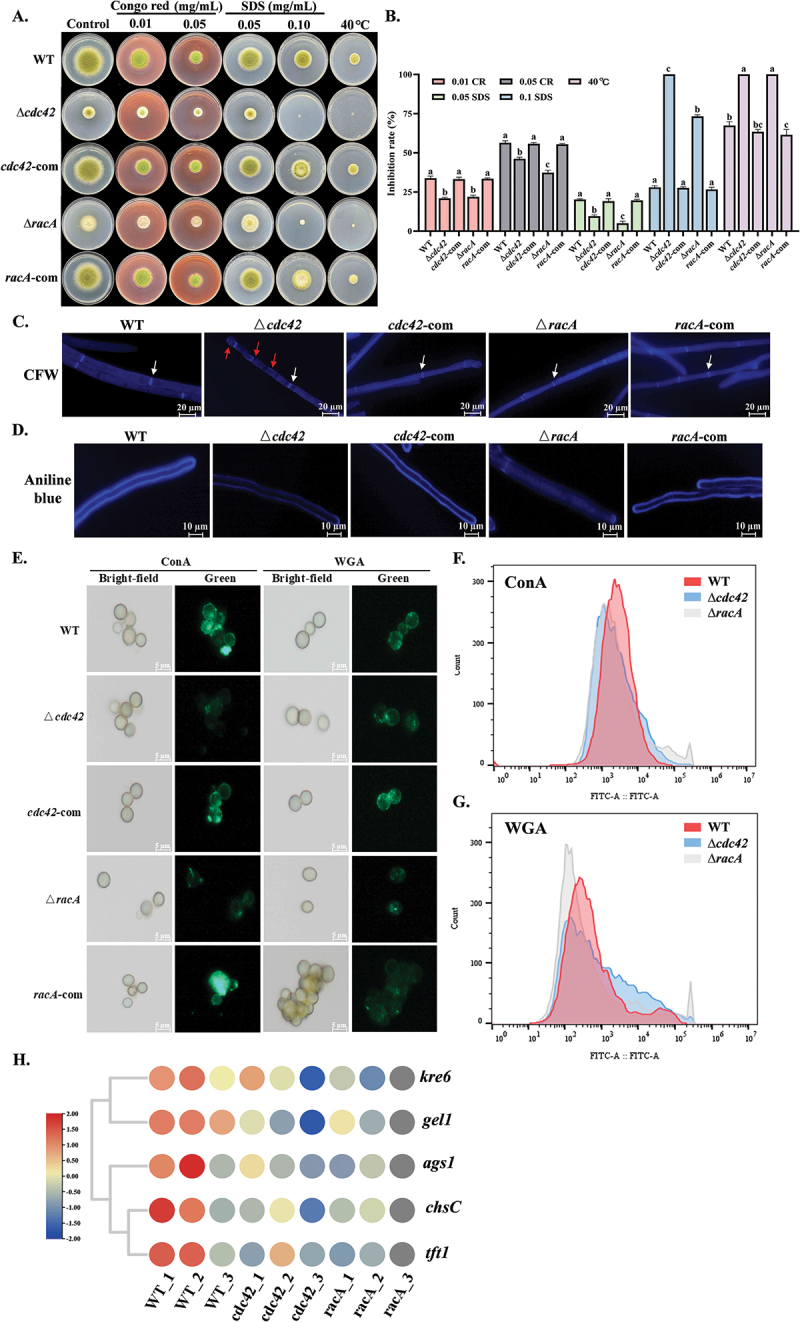
Effects of *cdc42* and *racA* on cell wall integrity. (A) Growth of all strains after 5 d of cultivation on GMM medium supplemented with CR and SDS at 30 °C. (B) Growth inhibition rates were calculated based on colony diameters. (C) Septum formation in all strains was analyzed using fluorescence microscopy. (D) β-1,3-glucan content in all strains was analyzed using fluorescence microscopy. (E) Chitin and mannan content in all strains were analyzed using fluorescence microscopy. (F, G) Chitin and mannan content in all strains were analyzed using flow cytometry. (H) RNA-seq analysis of the effects of *cdc42* and *racA* on the expression profiles of genes involved in cell wall biosynthesis. Error bars represent standard errors, and different letters above the bars indicate statistically significant differences (*p* < 0.05).

On GMM medium, the mutants exhibited abnormal sensitivity to high temperatures and were unable to grow at 40 °C (Figure 5A). When exposed to 0.1 mg/mL SDS, the inhibition rate of the mutants was significantly higher than that of the WT. The Δ*cdc42* strain demonstrated greater sensitivity than the Δ*racA* strain, with inhibition rates of 100% and 73.23%, respectively (Figure 5A,B). The deletion of *cdc42* and *racA* may result in defects in cell wall biosynthesis and modification. Although *cdc42* and *racA* did not have a significant impact on the septum (white arrows), slight chitin deposition (red arrows) was observed in the Δ*cdc42* mutant (Figure 5C). Analysis of cell wall components revealed that the contents of β-1,3-glucan, chitin, and mannan were reduced in the mutants (Figure 5D,E). Flow cytometric analysis revealed a decrease in fluorescence intensity in the mutants, suggesting alterations in cell wall composition (Figure 5F,G). Additionally, RNA sequencing revealed that the chitin synthase *chsC*, the β-1,3;1,4-glucan synthase gene *tft1*, the α-1,3-glucan synthase *ags1*, the β-1,3-glucanotransferase *gel1*, and the β-1,6-glucan gene *kre6* were down-regulated to varying degrees in the mutants (Figure 5H).

### 3.7. *Transcriptome analysis of WT, Δ*cdc42, *and Δ*racA *mutants*

**Figure 6.**
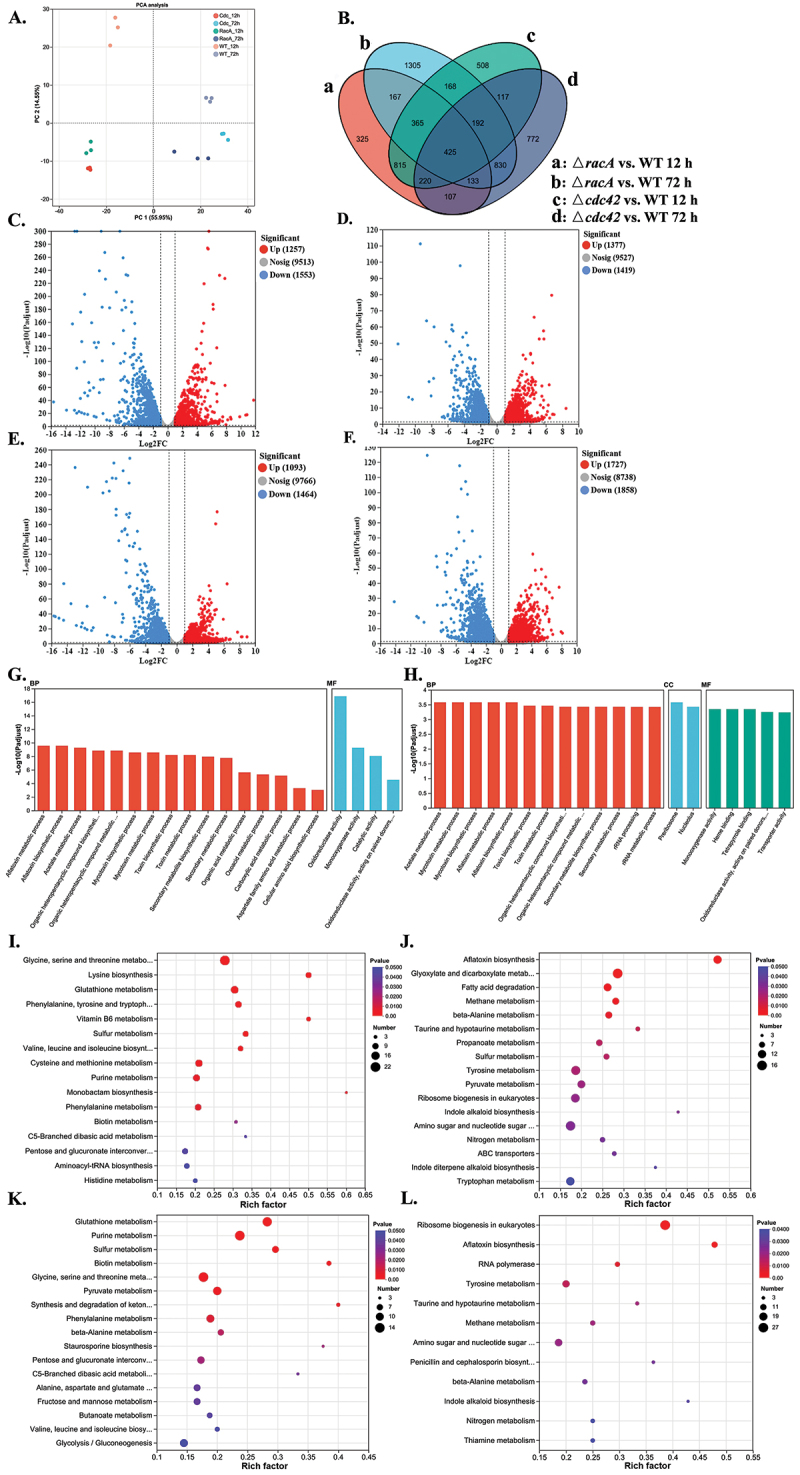
Transcriptomic analysis of WT and mutants. (A) Principal component analysis (PCA) plots for WT and mutants at 12 h and 72 h. (B) Venn diagram of all DEGs. (C, D) volcano plots of DEGs for Δ*cdc42* vs. WT at 12 h and 72 h. (E, F) Volcano plots of DEGs for Δ*racA* vs. WT at 12 h and 72 h. (G, H) GO enrichment analysis of DEGs for Δ*cdc42* vs. WT and Δ*racA* vs. WT. (I, J) KEGG enrichment of upregulated and downregulated DEGs for Δ*cdc42* vs. WT at 12 h. (K, L) KEGG enrichment of upregulated and downregulated DEGs for Δ*racA* vs. WT.

To explore the conserved and distinct regulatory functions of *cdc42* and *racA* in *A. flavus*, RNA was extracted from the WT, Δ*cdc42*, and Δ*racA* mutants at 12 and 72 h for transcriptome analysis. Principal component analysis demonstrated that samples from the same group clustered closely together, exhibiting strong parallels (Figure 6A). According to the TPM value, with a fold change of ≥ 2 as the threshold, a total of 2,810 differentially expressed genes (DEGs) were identified in the transcriptome comparison between Δ*cdc42* and WT at 12 h, and 2,796 DEGs were identified at 72 h (Figure 6C,D). At the same time, a total of 2,557 DEGs were identified between Δ*racA* and WT at 12 h, and 3,585 DEGs were identified at 72 h (Figure 6E,F). The regulatory effect of *racA* on *A. flavus* might be more extensive. The Venn diagram showed that Δ*cdc42* and Δ*racA* shared 815 and 830 DEGs at 12 and 72 h, respectively, indicating that the two play overlapping roles in cell regulation. However, the Δ*cdc42* uniquely regulated 508 and 772 DEGs at two different time points, and Δ*racA* uniquely regulated 325 and 1,305 DEGs, indicating that although the functions of the two are very similar, they still have differences in regulating *A. flavus* (Figure 6B). Gene ontology (GO) analysis showed that the DEGs of Δ*cdc42* and Δ*racA* were both involved in molecular functions (MF) and biological processes (BP), and *racA* was also involved in cellular components (CC) (Figure 6G,H). Pathway enrichment analysis of DEGs was performed using the KEGG database. At 12 h, the upregulated DEGs of Δ*cdc42* were significantly enriched in pathways related to amino acid synthesis and metabolism, vitamin B6 metabolism, sulfur metabolism, purine metabolism, and aminoacyl-tRNA biosynthesis. The downregulated DEGs were significantly enriched in aflatoxin synthesis, glyoxylate and dicarboxylic acid metabolism, fatty acid degradation, ABC transporters, nitrogen metabolism, pyruvate metabolism, ribosome biogenesis, and other pathways (Figure 6I,J). For Δ*racA*, the upregulated DEGs were significantly enriched in pathways related to amino acid synthesis and metabolism, sulfur metabolism, purine metabolism, pyruvate metabolism, ketone body synthesis and degradation, glycolysis and other pathways. The downregulated DEGs were significantly enriched in ribosome biogenesis, aflatoxin biosynthesis, RNA polymerase, and other pathways (Figure 6K,L). At 72 h, *cdc42* and *racA* continued to be involved in numerous primary and secondary metabolic processes in *A. flavus* (Figure S5A−D).

### 3.8. *Effect of* cdc42 *and* racA *on energy metabolism*

**Figure 7.**
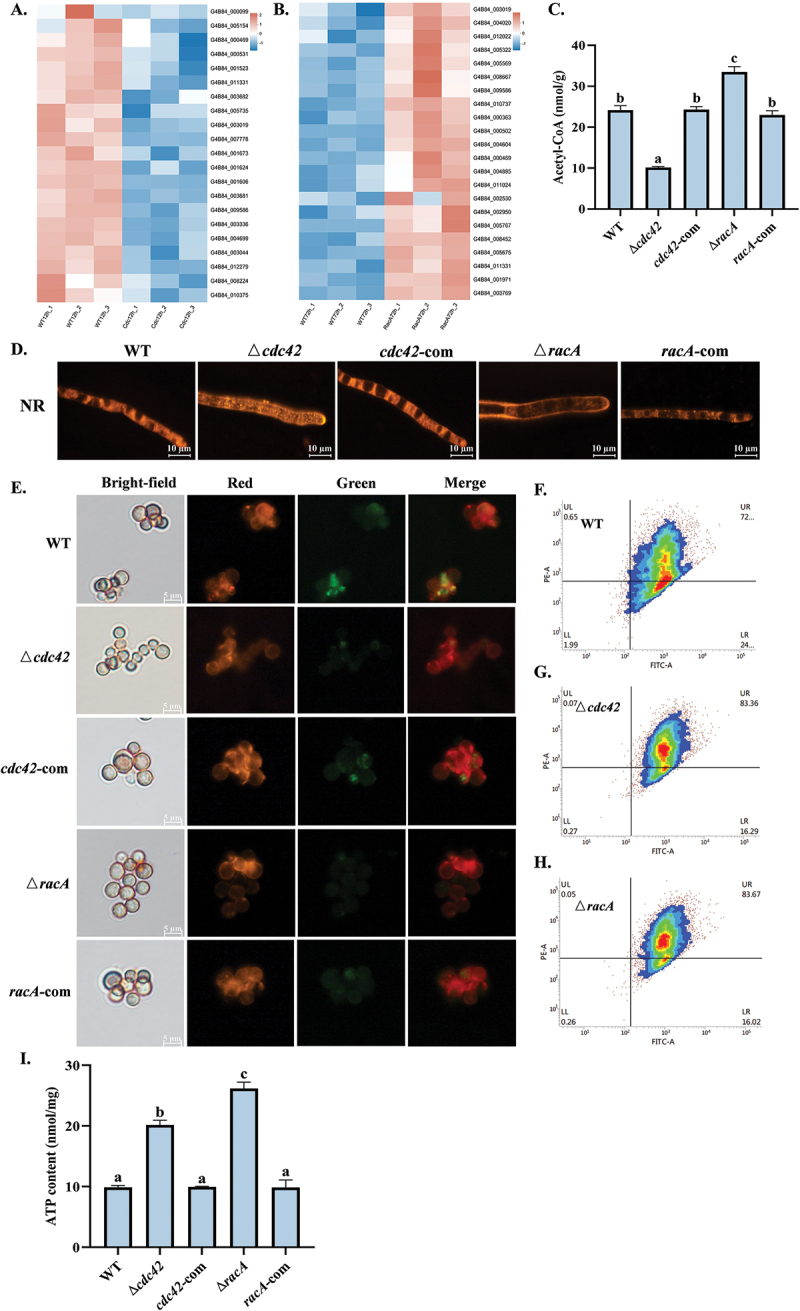
Effects of *cdc42* and *racA* on energy metabolism. (A) The downregulated DEGs related to fatty acid β-oxidation and pyruvate metabolism in Δ*cdc42* mutant. (B) The upregulated DEGs related to fatty acid β-oxidation and pyruvate metabolism in Δ*racA* mutant. (C) Acetyl-CoA content in all strains after cultivation at 30 °C for 72 h. (D) Lipid droplet accumulation in all strains after cultivation at 30 °C for 16 h in PDB medium. (E) Fluorescence microscopy analysis of mitochondrial membrane potential in all strains. (F–H) Flow cytometric analysis of mitochondrial membrane potential in WT, Δ*cdc42*, and Δ*racA* strains. (I) ATP content in all strains after cultivation at 30 °C for 72 h. Error bars represent standard errors, and different letters above the bars indicate statistically significant differences (*p* < 0.05).

Transcriptome analysis revealed that *cdc42* and *racA* play crucial roles in regulating energy metabolism. Acetyl-CoA is primarily produced through fatty acid β-oxidation and pyruvate metabolism (Ding et al. 2023). In the Δ*cdc42* mutant, genes associated with fatty acid degradation and pyruvate metabolism were significantly downregulated at 12 h (Figure 7A). Conversely, in the Δ*racA* mutant, genes related to pyruvate degradation and fatty acid degradation were significantly upregulated at 72 h (Figure 7B). Experimental results demonstrated that, compared to the WT, the acetyl-CoA content in the Δ*cdc42* mutant increased, while that in the Δ*racA* mutant decreased (Figure 7C). Acetyl-CoA serves as a precursor for the synthesis of triglycerides. Nile red staining revealed significant LD accumulation in the Δ*cdc42* mutant, whereas LD accumulation was notably reduced in the Δ*racA* mutant (Figure 7D). Both glycolysis and oxidative phosphorylation are critical pathways for ATP synthesis in cells. In the Δ*cdc42* mutant, genes associated with oxidative phosphorylation were significantly upregulated at 72 h (Figure S5E), whereas in the Δ*racA* mutant, genes related to pyruvate metabolism and glycolysis were significantly upregulated at 12 h (Figure S5F). To further investigate the effects of these two genes on energy metabolism, we assessed mitochondrial membrane potential (Δψm) using JC-1 staining. The mutants exhibited strong red fluorescence and weak green fluorescence, indicating an increase in Δψm, which was corroborated by flow cytometry analysis (Figure 7E−H). Subsequent detection of ATP content revealed that ATP levels were elevated in the mutants (Figure 7I).

### 3.9. *Effect of* cdc42 *and* racA *on lipid utilization and hydrolytic activity*

**Figure 8.**
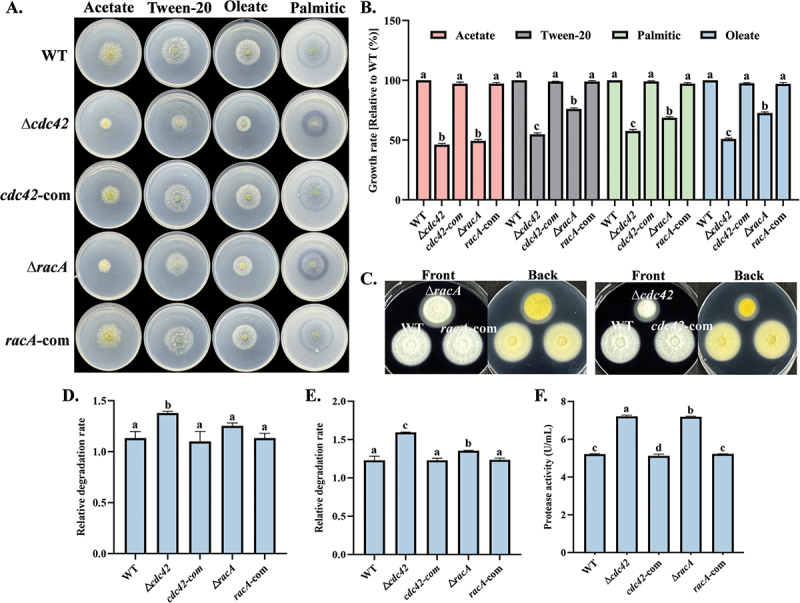
Effects of *cdc42* and *racA* on lipid utilization and hydrolytic activity. (A) Growth of all strains after 5 d of cultivation at 30 °C on MM medium supplemented with different fatty acids as sole carbon sources. (B) Growth rates were calculated based on colony diameters. (C) Starch degradation zones produced by all strains after 3 d of cultivation at 30 °C on AMM medium. (D) Starch degradation rates of all strains. (E) Fatty acid degradation rates of all strains after 5 d of cultivation at 30 °C on MM medium supplemented with 2.5 mmol/L palmitic acid. (F) Protease activity of all strains. Error bars represent standard errors, and different letters above the bars indicate statistically significant differences (*p* < 0.05).

*A*. *flavus* was spotted on MM medium containing various fatty acids as carbon sources. The growth rate of the mutants was significantly lower than that of the WT, while the ability of the Δ*racA* mutant to utilize lipids was notably superior to that of the Δ*cdc42* mutant (Figure 8A,B). By measuring the clearing zones on plates supplemented with palmitic acid and starch, we observed increased lipase and amylase activities in the mutants, along with calculated degradation rates (Figure 8C−E). Additionally, protease activity was evaluated using bovine serum albumin (BSA) as a substrate, revealing a significant increase in protease activity (Figure 8F).

## 4. Discussion

*A*. *flavus* is an opportunistic pathogen with significant impacts on agriculture, the economy, the environment, and human health. There is an urgent need to identify targets that can mitigate the survival, proliferation, and synthesis of toxic compounds produced by *A. flavus* (Lohmar et al. 2019). In this study, we investigated the roles of *cdc42* and *racA* in the morphogenesis, reproduction, aflatoxin synthesis, and pathogenicity of *A. flavus*. The two genes exhibit considerable functional redundancy, and the loss of both functions is lethal. However, there are significant differences in their functional deployment.

In *A. niger*, RacA regulates cell polarity, while the loss of Cdc42 has minimal effects on growth and does not alter hyphal morphology (Kwon et al. 2011). In *A. nidulans*, RacA deficiency leads to growth defects, whereas Cdc42 plays a dominant role in polarized hyphal growth (Virag et al. 2007). In *Ustilago maydis*, Rac1 is crucial for polarized hyphal formation, while Cdc42 regulates septum formation (Mahlert et al. 2006). Based on observations of nuclear distribution, SRD, and septa, we propose that in *A. flavus*, Cdc42 regulates hyphal extension, whereas RacA influences cell polarity. However, neither impacts septum formation. In spore polarization, the loss of either *cdc42* or *racA* leads to delayed spore polarization in *A. niger* (Kwon et al. 2011) and *A. nidulans* (Virag et al. 2007). However, in *A. flavus*, the loss of *cdc42* and *racA* significantly increases the spore germination rate, suggesting that *cdc42* and *racA* negatively regulate the spore polarization process, which is consistent with the findings in *A. fumigatus* (Li et al. 2011). The accelerated conidial germination rate also brings about high energy demands, increasing the production and secretion of extracellular hydrolases, and the ATP content in the mutant is significantly increased. The loss of *cdc42* and *racA* disrupts the homeostasis during spore germination, resulting in malformed conidia and a sharp decrease in number. In addition, sclerotia were not observed in the mutant, indicating that *cdc42* and *racA* positively regulate the reproductive process of *A. flavus*. The fungal cell wall is a conserved structure primarily composed of chitin, mannan, and glucan (Sun et al. 2018). This structure is essential for growth, morphogenesis, and stress responses (Gow and Lenardon 2023). The mutants exhibited an inability to grow on GMM medium at 40 °C, likely due to cell wall defects that increase sensitivity to high temperatures. This hypothesis was supported by the high sensitivity of the mutants to cell wall disruptors SDS. RNA sequencing revealed that the chitin synthase *chsC*, the β-1,3;1,4-glucan synthase gene *tft1*, the α-1,3-glucan synthase *ags1*, the β-1,3-glucanotransferase *gel1*, the β-1,6-glucan gene *kre6*, and the GPI-anchored CFEM domain protein CfmA were all down-regulated to varying degrees in the mutants. The expression of *cfmA* was reduced by 8.13-fold and 10.5-fold compared to that of the WT, which subsequently affected glucan assembly (Samalova et al. 2020). Fluorescence microscopy and flow cytometry analyses demonstrated that the deletion of *cdc42* and *racA* resulted in significant alterations to the cell wall composition of *A. flavus*.

Biosynthesis of aflatoxin is a tightly regulated process involved in a complex, interconnected network, and approximately 30 genes in the cluster have been identified (Tumukunde et al. 2021). In the Δ*cdc42* and Δ*racA* mutants, 21 genes were significantly down-regulated. Among them, *aflR* and *aflS* are the two most important specific regulatory genes. The *aflS* gene interacts with *aflR* to modulate the transcription of the aflatoxin pathway and is essential for activating *aflR* (Georgianna and Payne 2009). At least 17 genes in the cluster have AflR binding sites (Shimizu et al. 2003). When *aflS* is downregulated, *aflR* activation is hindered, which may affect the activation expression of some genes. The expression levels of *aflA*, *aflC*, and *hypC* were significantly downregulated, impacting the elongation of the polyketide backbone and subsequent conversion to norsolorinic acid (NOR). At 12 h, *aflD*, *aflE*, *aflH*, *aflK*, and *aflV* were almost not expressed in the mutants, resulting in the obstruction of the conversion of NOR to versicolorin A (VERA) (Caceres et al. 2020). Among them, the downregulation of *aflK* affected the closure of the furan ring and DNA binding (Yu et al. 2004). In the mutants, the conversion of VERA to sterigmatocystin (ST) was strongly inhibited due to the notable downregulation of four crucial genes involved in this pathway, specifically *aflN*, *aflY*, *aflX*, and *aflO*. ST is the penultimate intermediate of the AFB_1_ biosynthesis pathway. The *aflQ* gene encodes an oxidoreductase that participates in the conversion of O-methylsterigmatocystin (OMST) to AFB_1_ and dihydro-O-methylsterigmatocystin (DHOMST) to AFB_2_. Its expression level has a strong linear relationship with the biosynthesis of aflatoxin (Yang et al. 2019). The expression level of *aflQ* in the mutants is extremely low, which affects the final formation of AFB_1_. Oxidative reactions involved in the aflatoxin biosynthetic pathway represent an additional source of intracellular ROS (Tian et al. 2021). KA, a potent antioxidant that scavenges ROS, is inversely correlated with aflatoxin levels (Zhang et al. 2014). In the mutants, aflatoxin production was significantly reduced, while KA levels were elevated, suggesting that ROS accumulation may be lower in the mutant compared to the WT.

The regulation of ROS by *cdc42* and *racA* in fungi varies across different systems. In *Epichloë festucae*, *cdc42* and *racA* have opposing roles in controlling ROS production (Kayano et al. 2018). In *A. nidulans*, *racA* is believed to activate Nox to generate ROS, while *cdc42* is thought to regulate Nox localization. The loss of either protein results in a decrease in ROS levels (Virag et al. 2007). Similar conclusions were observed in *A. flavus*, where the loss of *cdc42* and *racA* also resulted in reduced levels of ROS. These proteins regulate ROS production by interacting with NoxR, the regulatory subunit of NADPH oxidase (Nox). The loss of *cdc42* and *racA* also led to decreased activity of antioxidant enzymes (SOD and CAT). After *A. flavus* infects its host, the host employs various defense mechanisms, resulting in the rapid accumulation of ROS at the infection site (Liu et al. 2010). To counteract the adverse effects of ROS produced by the host, *A. flavus* employs a ROS scavenging system to eliminate excess ROS, thereby enhancing its survival under stress (Zhang et al. 2018). In the mutants, the balance of the oxidative defense system in *A. flavus* was disrupted. Fungal attachment to surfaces is crucial for biofilm formation and evasion of the host defense mechanisms (Flemming and Wingender 2010). At 48 h, the attachment ability of the mutant was significantly impaired. Furthermore, the cytoskeleton of mutants was severely damaged, lacking the mechanical force necessary to penetrate host tissue (Qin et al. 2022). These factors profoundly impacted the colonization of *A. flavus* within the host during the early stages of infection.

Cdc42 and RacA are implicated in the energy metabolism of *A. flavus*. In Δ*cdc42* mutant, genes associated with oxidative phosphorylation are significantly upregulated, resulting in a short-term increase in ATP content. Acetyl-CoA is primarily generated through fatty acid β-oxidation and pyruvate metabolism pathways (Maggio-Hall et al. 2005; Ding et al. 2023). Regarding lipid and pyruvate metabolism, Cdc42 plays a negative regulatory role, while RacA exerts a positive regulatory function. Compared to the WT, Δ*cdc42* mutant mycelia exhibited increased LDs accumulation but decreased acetyl-CoA levels. Acetyl-CoA, a precursor for polyketide synthesis, is positively correlated with aflatoxin production (Yu et al. 2004; Fountain et al. 2016). The disruption of fatty acid β-oxidation and pyruvate metabolism pathways in Δ*cdc42* mutant impairs the accumulation of precursors required for aflatoxin synthesis. The fatty acid β-oxidation and pyruvate metabolism pathways were upregulated in the Δ*racA* mutant, leading to enhanced fatty acid utilization and reduced LDs accumulation. Despite the growth defect inherent to the *racA* gene, the growth rate of the Δ*racA* mutant was significantly higher than that of the Δ*cdc42* mutant when grown on media containing short-, medium-, and long-chain fatty acids. Furthermore, glycolysis was significantly upregulated in the Δ*racA* mutant compared with the WT, which may promote the accumulation of acetyl-CoA. The elevated acetyl-CoA content provides the necessary precursors for the accelerated operation of the TCA cycle. Consequently, the energy metabolism rate in the Δ*racA* mutant is enhanced, resulting in increased ATP production. Transcriptome analysis revealed that *cdc42* and *racA* exhibit overlapping functions and further regulate various biological processes, including amino acid metabolism, purine metabolism, ribosome biogenesis, nitrogen metabolism, sulfur metabolism, and DNA replication. These metabolic pathways not only maintain cellular nutrient homeostasis but also support growth, development, and responses to environmental stress. Thus, *cdc42* and *racA* play key roles in reshaping metabolic processes.

This study systematically elucidated the extensive regulatory roles of the closely related *cdc42* and *racA* genes in controlling the reproductive capacity, morphogenesis, oxidative stress response, aflatoxin synthesis, and pathogenicity of *A. flavus*. Our results reveal a unique regulatory pattern of *cdc42* and *racA* in *A. flavus* and provide insights into the prevention and control of aflatoxin contamination.

## Supplementary Material

0626-revised-Supplementary_Materials_Clean.docx
