# Supplementary material for: Functionally redundant Rho GTPases Cdc42 and RacA regulate aflatoxin synthesis and pathogenicity in Aspergillus flavus by controlling morphogenesis, oxidative balance and energy metabolism
Source: Mycology. 2025 Jul 10;17(1):2527381. doi: 10.1080/21501203.2025.2527381 (PMC13007405; doi:10.1080/21501203.2025.2527381)
Supplement: 0626-revised-Supplementary_Materials_Clean.docx [file TMYC_A_2527381_SM8583.docx]

Table S1. All mutants constructed in this study.

| **Genotype/**  **Background** | **Target gene** | **Protospacer sequence (NGG)** | **(Mutants/Transformants)** |
| --- | --- | --- | --- |
| Δ*wA*  (PU6-500) | *wA* | GTGGATCTACTGGCGCGTCAC**CGG** | 15/16 14/16 15/16 |
| Δ*yA*  (PU6-500) | *yA* | CGCCAAATGATTCTCACTAA**TGG** | 9/9 15/16 15/16 |
| Δ*wA*  (PU3-500) | *wA* | GTGGATCTACTGGCGCGTCAC**CGG** | 10/11 10/12 13/15 |
| Δ*yA*  (PU3-500) | *yA* | CGCCAAATGATTCTCACTAA**TGG** | 15/16 13/15 12/15 |
| Δ*wA*  (P5S rRNA-500) | *wA* | GTGGATCTACTGGCGCGTCAC**CGG** | 15/16 8/9 15/15 |
| Δ*yA*  (P5S rRNA-500) | *yA* | CGCCAAATGATTCTCACTAA**TGG** | 14/15 13/13 15/16 |
| Δ*wA*  (P5S rRNA-350) | *wA* | GTGGATCTACTGGCGCGTCAC**CGG** | 14/15 11/12 16/16 |
| Δ*yA*  (P5S rRNA-350) | *yA* | CGCCAAATGATTCTCACTAA**TGG** | 16/16 15/16 15/16 |
| Δ*wA*  (P5S rRNA-116) | *wA* | GTGGATCTACTGGCGCGTCAC**CGG** | 13/14 15/16 15/15 |
| Δ*yA*  (P5S rRNA-116) | *yA* | CGCCAAATGATTCTCACTAA**TGG** | 15/16 12/13 15/16 |
| Δ*wA/pyrG*  (tRNA^Gly^) | *wA, pyrG* | GTGGATCTACTGGCGCGTCAC**CGG**  GTGGATTATATAACAGGACT**CGG** | 12/13 10/11 9/9 **^a^**  9/13 8/11 8/9 ^b^ |
| Δ*wA/ pyrG* (tRNA^Ile^) | *wA, pyrG* | GTGGATCTACTGGCGCGTCAC**CGG**  GTGGATTATATAACAGGACT**CGG** | 10/10 7/8 12/13 **^a^**  9/10 6/8 8/13 ^b^ |
| Δ*wA/ pyrG* (tRNA^Arg^) | *wA, pyrG* | GTGGATCTACTGGCGCGTCAC**CGG**  GTGGATTATATAACAGGACT**CGG** | 25/26 15/16 9/10 **^a^**  20/26 10/16 6/10 ^b^ |
| Δ*wA/ pyrG* (tRNA^Arg+Ile+Gly^） | *wA, pyrG* | GTGGATCTACTGGCGCGTCAC**CGG**  GTGGATTATATAACAGGACT**CGG** | 9/9 14/15 15/16 **^a^**  7/9 12/15 12/16 ^b^ |
| Δ*cdc42* | *cdc42* | CACAACAAACAAGTTCCCCT**CGG** | 7/9 8/9 |
| Δ*racA* | *racA* | CAAAGATTGGGTAGCGGGAC**CGG** | 6/8 6/9 |
| Δ*cdc42/racA* | *cdc42, racA* | CACAACAAACAAGTTCCCCT**CGG**  CAAAGATTGGGTAGCGGGAC**CGG** | 7/12 5/12 7/16 ^c^  3/12 5/12 5/16 ^d^  0/12 0/12 0/16 ^b^ |
| *racA-*com | - | - | - |
| *cdc42*-com | - | - | - |
| Δ*cdc42racA_tetOn_* | - | - | - |

^a^ The number of Δ*wA* mutants in transformants.

^b^ The number of double-gene mutants in transformants.

^c^ The number of Δ*cdc42* mutants in transformants.

^d^ The number of Δ*racA* mutants in transformants.

Table S2. Primer sequences in this study.

| **Primer** | **Sequence (5’-3’)** |
| --- | --- |
| TU6-F | ACATATTTAAAAAAAGTCT |
| scaffold-R | GTTTTAGAGCTAGAAATAGC |
| P5SrRNA-F | TATTTCTAGCTCTAAACGTGACGCGCCAGTAGATCC |
| 5SrRNA-R | ATACGACCATAGGGTGTGGA |
| TU6OL-F | TGCAACCTAGTAACGGTACCACATATTTAAAAAAAGTCT |
| P5SrRNAOL-R | AACTAGGTCAAAATCTGCAGATACGACCATAGGGTGTGGAG |
| cdck-F | GATTGATGTCCCCAGCAGAATGAT |
| cdck-R | TCCTATGAAGGCGTCGGGTC |
| rack-F | GATCGATTCTTATAATTTTCTCCCGACT |
| rack-R | ACGACGGAGGGCTGACGATGGAG |
| cdc425-F | CTCCTCTTCTTCCCACTTCT |
| cdc42OL-R | GGCTCATCGTCACCCCATGCCCTTACAGCAAAACGCACTTCT |
| racA5-F | ATCGATTCTTATAATTTTCT |
| racAOL-R | GGCTCATCGTCACCCCATCTACAGAATCAGACATTTGC |
| ptrA-F | GGGCAATTGATTACGGGAT |
| ptrA-R | ATGGGGTGACGATGAGCCG |
| cdc423OL-F | TCCCGTAATCAATTGCCCTCGAACACGCATCTCTTTAG |
| cdc423-R | CCGGTGCGGAGTTAACCATGT |
| racA3OL-F | TCCCGTAATCAATTGCCCAGAACAGACAACCAGAATGAG |
| racA3-R | TCTTTCAGAATCTGCGATATAT |
| AP-R | ATTCTATCCGTTCCTTTTGCTCCT |
| BP-F | AAGGCTGCCGAGGAGGCAT |
| 18S rRNA-F | TGATGACCCGCTCGGCACCTTACGAGAAATCAAAGT |
| 18S rRNA-R | GGCCATGCACCACCATCCAAAAGATCAAGAAAGAGC |
| aflC-F | ACGGAATTTGGTCCCGATGG |
| aflC-R | GTCAGCATCCAGGTCCGTTC |
| aflD-F | CGCCTGAGGAGACGGTGTATT |
| aflD-R | CTGCCTTCAGCGACGGTTAG |
| aflR-F | AACAAGAGGGCTACCGATGC |
| aflR-R | TACCATGCCAGCACCTTGAG |
| aflS-F | ATGTGCGAATCCTATCCCCC |
| aflS-R | ACGAGGAAACGGAGTGATGG |
| aflO-F | CTTTCGGCAGTGACCTAACC |
| aflO-R | TCTTGAACTATAAGGCGACCA |
| aflP-F | ACGAAGCCACTGGTAGAGGAGAT |
| aflP-R | GTGAATGACGGCAGGCAGGT |
| aflQ-F | GTCGCATATGCCCCGGTCGG |
| aflQ-R | GGCAACCAGTCGGGTTCCGG |
| aflK-F | GAACTGCTTCAGTTGCCGTG |
| aflK-R | GCGGTGTGGTGTATTGATACC |
| cat1-F | CATTGCCAGTGCGGCGT |
| cat1-R | TCCGACAGAAGATCCAGCGCT |
| cat2-F | AGGACCAGACGGATGTCGAGT |
| cat2-R | CGTAGTTGGCATTCAGAGCCCG |
| sod1-F | TAACCCCCAACCGTCAAAAT |
| sod1-R | CTTCACAAGGTGCTCAAATCT |
| mnsod-F | TAAGCCTGGGGATTTACTGGT |
| mnsod-R | AAGTACCAGCAAGTACTATAG |
| hyr1-F | TCCCAGAAACCATTAATCTACT |
| hyr1-R | GAAGGTGGTTGCGGAAGCCATG |
| nox1-F | CTCACTGAGCATTCTTAGGTAT |
| Nox1-R | GGCCTGAGAGTTTTGACAATGT |

Table S3. Similarity and identity of RacA/Cdc42 between eukaryotic organisms.

| **Species** | **Length** | **Identity (%)**  **(Cdc42/RacA)** | **Similarity (%)**  **(Cdc42/RacA)** |
| --- | --- | --- | --- |
| *Aspergillus flavus* | 192/204 | 100/100 | 100/100 |
| *Aspergillus nidulans* | 192/199 | 95.8/90.7 | 97.9/93.1 |
| *Aspergillus fumigatus* | 192/199 | 97.4/93.6 | 98.4/95.6 |
| *Homo sapiens* | 191/192 | 79.4/76.8 | 88.4/88.9 |
| *Saccharomyces cerevisiae* | 191/- | 82.5/- | 89.9/- |
| *Neurospora crassa* | 196/200 | 79.8/78.6 | 88.3/88.5 |
| *Candida albicans* | 195/183 | 82.0/64.5 | 90.5/79.2 |
| *Penicillium digitatum* | 193/201 | 95.9/91.7 | 97.9/92.2 |
| *Danio rerio* | 191/192 | 79.4/75.8 | 87.3/88.9 |

-, no RacA homologues was detected in *Saccharomyces cerevisiae.*

A.

GAGAGAAACTGGACAGAGCTTCGAGAGAGAACTCGGCGTGAAAACGACGCCACCCTTATCACGCACCAGCCTTCTGGGCCGGCGGGACACAGGCGAGGACAGGCAAATTTAGAAATCGAGTTCGGTAGTCAAACCAGCTAGATAAATTCTCAGATTCTGGCCTCGGATGGTTGGACACATCGACTCTGATTTATTCTCTACCTCATGGTCTCAGGCTCGACTACAGCTTAGATGGTCTCGTATATAGATTCGATACAAGACGTCCCAACCATGCCACCTTGTTCATGTCGAGTACGCATTATAGCCGCGTTGCTTTCTTCTGAATCCTGATGAACATGTTTCTCTCTGAACCATGCATCAGCAATAGGAGCTTCTAGAGGCGCATCTCATTATACCAAGCGCAGCCAGTAGTCACGCAGACATTTGGGATTGTTGCTTGTATGACTAAATACAATATAGCATGGCTACTAGATAATCTACTACTGAAAAGTAACAGATACACATACGACCATAGGGTGTGGAGAACAGGGCTTCCCGTCCGCTCAGCCGTACTTAAGCCACACGCCGGGAGGTTAGTAGTTGGGTGGGTGACCACCAGCGAATCCCTCCTGTTGTATGT

5S rRNA (-500)/5S rRNA gene

B.

CAGATTCTGGCCTCGGATGGTTGGACACATCGACTCTGATTTATTCTCTACCTCATGGTCTCAGGCTCGACTACAGCTTAGATGGTCTCGTATATAGATTCGATACAAGACGTCCCAACCATGCCACCTTGTTCATGTCGAGTACGCATTATAGCCGCGTTGCTTTCTTCTGAATCCTGATGAACATGTTTCTCTCTGAACCATGCATCAGCAATAGGAGCTTCTAGAGGCGCATCTCATTATACCAAGCGCAGCCAGTAGTCACGCAGACATTTGGGATTGTTGCTTGTATGACTAAATACAATATAGCATGGCTACTAGATAATCTACTACTGAAAAGTAACAGATACACATACGACCATAGGGTGTGGAGAACAGGGCTTCCCGTCCGCTCAGCCGTACTTAAGCCACACGCCGGGAGGTTAGTAGTTGGGTGGGTGACCACCAGCGAATCCCTCCTGTTGTATGT

5S rRNA (-350)/5S rRNA gene

C.

GCGGGTGTAGTCTAGTGGTTATGATGCGCGCTTCCCAAGCGCGAGGTCCGGGGTTCGAACCCCCGTTCCCGCAGAAAGATGCCTCGCAGCTTATGTTTTAGAGCTAGAAATAGCAAGTTAAAATAAGGCTAGTCCGTTATCAACTTGAAAAAGTGGCACCGAGTCGGTGCGCGGGTGTAGTCTAGTGGTTATGATGCGCGCTTCCCAAGCGCGAGGTCCGGGGTTCGAACCCCCGTTCCCGCAAGAAACCATCACCGGTCTGAGTTTTAGAGCTAGAAATAGCAAGTTAAAATAAGGCTAGTCCGTTATCAACTTGAAAAAGTGGCACCGAGTCGGTGCGCGGGTGTAGTCTAGTGGTTATGATGCGCGCTTCCCAAGCGCGAGGTCCGGGGTTCGAACCCCCGTTCCCGCA

tRNA^Gly^-protospacer(*wA*)-scaffold-tRNA^Gly^-protospacer(*pyrG)*-scaffold-tRNA^Gly^

D.

GGTCCCATAACTCAGTTGGTTAGAGTGCTGAGCTAATAACTCAGAAGTCGAGAGTTCGAGCCTCCCTGGGACCAGAAAGATGCCTCGCAGCTTATGTTTTAGAGCTAGAAATAGCAAGTTAAAATAAGGCTAGTCCGTTATCAACTTGAAAAAGTGGCACCGAGTCGGTGCGGTCCCATAACTCAGTTGGTTAGAGTGCTGAGCTAATAACTCAGAAGTCGAGAGTTCGAGCCTCCCTGGGACCAAGAAACCATCACCGGTCTGAGTTTTAGAGCTAGAAATAGCAAGTTAAAATAAGGCTAGTCCGTTATCAACTTGAAAAAGTGGCACCGAGTCGGTGCGGTCCCATAACTCAGTTGGTTAGAGTGCTGAGCTAATAACTCAGAAGTCGAGAGTTCGAGCCTCCCTGGGACCA

tRNA^Ile^-protospacer(*wA*)-scaffold-tRNA^Ile^-protospacer(*pyrG)*-scaffold-tRNA^Ile^

E.

GGCCGGCTGGCCCAATGGCAAGGCGCTTGACTACGAATCAAGAGATTGCAGGTTCGACCCCTGCGTCGGTCAGAAAGATGCCTCGCAGCTTATGTTTTAGAGCTAGAAATAGCAAGTTAAAATAAGGCTAGTCCGTTATCAACTTGAAAAAGTGGCACCGAGTCGGTGCGGCCGGCTGGCCCAATGGCAAGGCGCTTGACTACGAATCAAGAGATTGCAGGTTCGACCCCTGCGTCGGTCAAGAAACCATCACCGGTCTGAGTTTTAGAGCTAGAAATAGCAAGTTAAAATAAGGCTAGTCCGTTATCAACTTGAAAAAGTGGCACCGAGTCGGTGCGGCCGGCTGGCCCAATGGCAAGGCGCTTGACTACGAATCAAGAGATTGCAGGTTCGACCCCTGCGTCGGTCA

tRNA^Arg^-protospacer(*wA*)-scaffold-tRNA^Arg^-protospacer(*pyrG*)-scaffold-tRNA^Arg^

F.

GCGGGTGTAGTCTAGTGGTTATGATGCGCGCTTCCCAAGCGCGAGGTCCGGGGTTCGAACCCCCGTTCCCGCAGAAAGATGCCTCGCAGCTTATGTTTTAGAGCTAGAAATAGCAAGTTAAAATAAGGCTAGTCCGTTATCAACTTGAAAAAGTGGCACCGAGTCGGTGCGGCCGGCTGGCCCAATGGCAAGGCGCTTGACTACGAATCAAGAGATTGCAGGTTCGACCCCTGCGTCGGTCAAGAAACCATCACCGGTCTGAGTTTTAGAGCTAGAAATAGCAAGTTAAAATAAGGCTAGTCCGTTATCAACTTGAAAAAGTGGCACCGAGTCGGTGCGGTCCCATAACTCAGTTGGTTAGAGTGCTGAGCTAATAACTCAGAAGTCGAGAGTTCGAGCCTCCCTGGGACCA

tRNA^Gly^-protospacer(*wA*)-scaffold-tRNA^Arg^-protospacer(*pyrG*)-scaffold-tRNA^Ile^

**Figure S1.** *5S rRNA* gene and its upstream 350 bp (A) and 500 bp (B) sequences in *Aspergillus flavus* NRRL3357. Sequences of tRNA-gRNA tandem arrays using individual glycine (C), isoleucine (D), arginine (E), and a mix of the three amino acids (F).


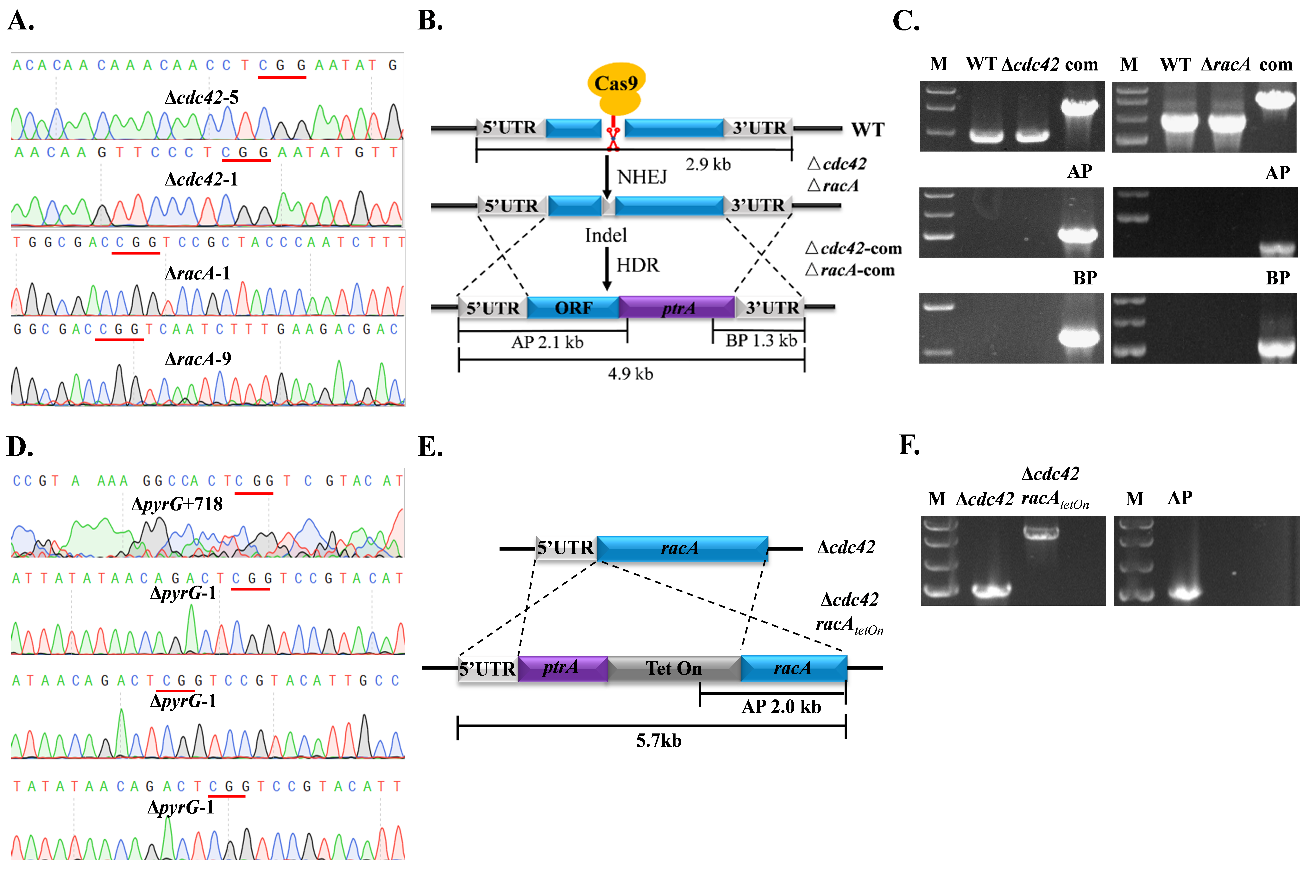


**Figure S2.** (A) Nucleotide sequence analysis of target sequences in Δ*cdc42* and Δ*racA* strains, with the PAM sequence indicated by the red underline. 5-bp and 1-bp deletions were detected in the Δ*cdc42* strain, and 1-bp and 9-bp deletions were detected in the Δ*racA* strain. (B) Strategy for constructing complementation strains using HR. (C) PCR analysis using genomic DNA as a template to verify the complementation of the strains. AP contains 5'UTR, ORF, and part of *ptrA*. BP contains part of *ptrA* and 3'UTR. M: marker; lane 1: WT; lane 2: Δ*cdc42*/Δ*racA*; lane 3: *cdc42*-com/*racA*-com. (D) Nucleotide sequence analysis of target sequences in the Δ*wA*/*pyrG* double-gene mutant, with the PAM sequence indicated by the red underline. 11-bp deletions and 311-bp insertions were detected for the *wA* gene, and 1-bp deletion and 718-bp insertions were detected for the *pyrG* gene. (E) Strategy for constructing doxycycline-inducible Δ*cdc42racA_tetOn_* mutant using HR. (F) PCR analysis using genomic DNA as a template to verify the Δ*cdc42racA_tetOn_* mutant. AP contains ORF, and part of Tet-On promoter. M: marker; lane 1: Δ*cdc42*; lane 2: Δ*cdc42racA_tetOn_*_._


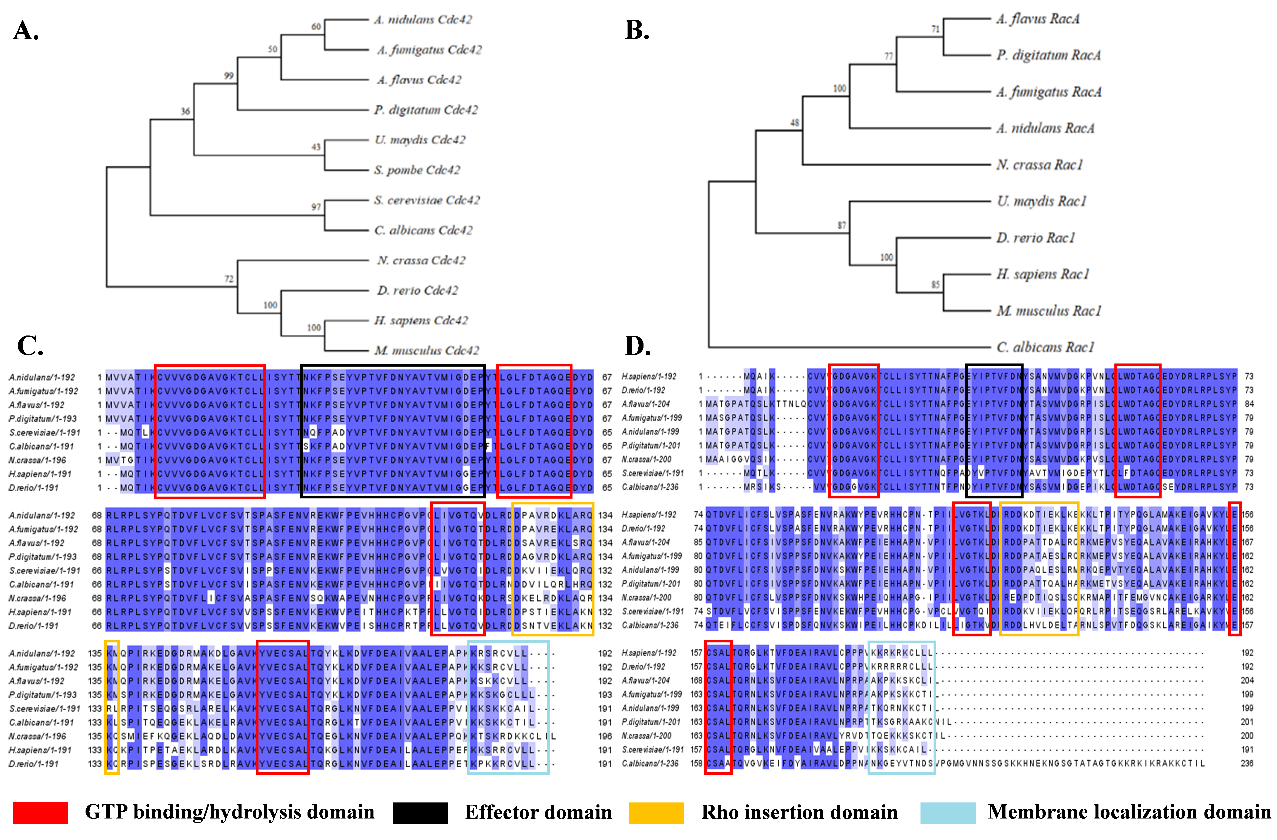


**Figure S3.** Identification of Cdc42 and RacA proteins in *Aspergillus flavus*. Phylogenetic tree analysis of Cdc42 (A) and RacA (B) in different eukaryotes. Analysis of amino acid conservation and functional domains of putative Cdc42 (C) and RacA (D) in different eukaryotes. GTP binding/hydrolysis domain (red box), effector domain (black box), Rho insertion domain (yellow box), and membrane localization domain (blue box).


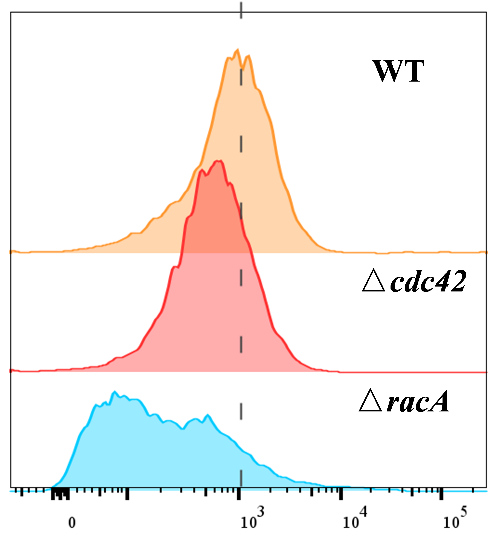


**Figure S4.** Flow cytometric analysis of ROS in WT, Δ*cdc42*, and Δ*racA* strains.


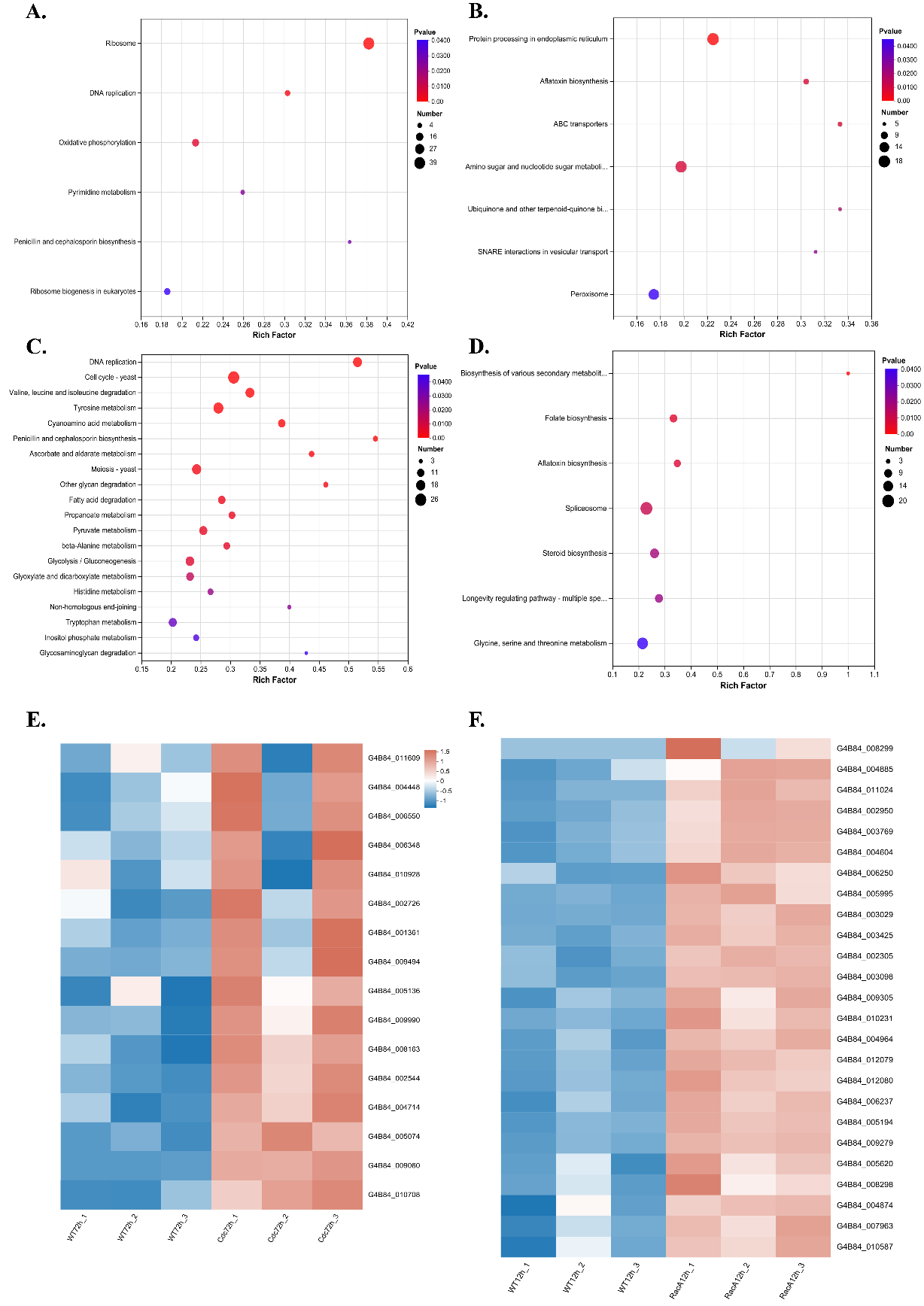
**Figure S5.** KEGG enrichment of upregulated (A) and downregulated (B) DEGs for Δ*cdc42* vs. WT at 72 h. KEGG enrichment of up-regulated (C) and down-regulated (D) DEGs for Δ*racA* vs. WT at 72 h. (E) Upregulated DEGs associated with oxidative phosphorylation metabolism in the Δ*cdc42* mutant. (F) Upregulated DEGs associated with pyruvate metabolism and glycolysis in the Δ*racA* mutant.
